# Personalized and differentially private federated learning for mobility data mining via the TrajectoryGuard framework

**DOI:** 10.1371/journal.pone.0358703

**Published:** 2026-10-29

**Authors:** Ali Alqazzaz

**Affiliations:** Department of Information Systems and Cybersecurity, College of Computing and Information Technology, University of Bisha, Bisha, Saudi Arabia; Beijing Technology and Business University, CHINA

## Abstract

This paper presents **TrajectoryGuard**, a personalized and privacy-preserving federated learning framework for secure and efficient mobility data mining. The framework integrates differential privacy, secure multiparty aggregation, and adaptive gradient compression to ensure that raw location traces, such as GPS logs or geo-tagged user activities, remain confined to user devices, thereby minimizing privacy leakage risks. Unlike conventional approaches, TrajectoryGuard introduces a dynamic noise-aware optimization mechanism and personalized aggregation scheme to enhance both model accuracy and robustness under non-IID and heterogeneous client scenarios. Experimental evaluation on the GeoLife dataset and the T-Drive Beijing taxi trajectory dataset demonstrates competitive performance, achieving 92.5% accuracy and 30% reduction in total per-round communication overhead (comprising gradient payload, model broadcast, and encryption metadata) while maintaining strong privacy guarantees (ε = 0.9 at σ^2^ = 1.0). Comparative results against FedAvg, FedProx, AdaptFL, and Fed-Ensemble confirm TrajectoryGuard’s superiority in convergence stability, privacy resilience, and predictive utility. The framework’s practical viability is illustrated through a real-world case study in urban trajectory forecasting, highlighting its relevance for applications in smart transportation and behavior modeling. The accuracy metric specifically corresponds to next-destination classification, a multi-class problem where the model predicts a destination category (e.g., home, workplace, or shopping location) from partial GPS trajectories. Evaluation follows an 80/20 time-based train/test split per user to prevent data leakage across temporal boundaries.

## 1. Introduction

Mobility data mining is pivotal in modern applications such as intelligent transportation systems, urban planning, and location-based services. By analyzing mobility patterns, organizations can derive insights into traffic flow, user behavior, and service optimization [1,2]. However, mobility data often contains sensitive personal information, including location trajectories and timestamps, making privacy preservation a critical concern [3,4]. Traditional centralized approaches require collecting raw trajectory data on a central server. While effective for model performance, these approaches expose sensitive user information to risks such as data breaches, unauthorized access, and misuse. These concerns have drawn increasing attention and prompted the adoption of privacy-preserving techniques, further reinforced by legal mandates like the General Data Protection Regulation (GDPR) and the California Consumer Privacy Act (CCPA). To address these challenges, privacy-aware mobility data mining frameworks have been developed to preserve data utility while protecting user confidentiality [5,6]. Among them, federated learning (FL) has emerged as a promising solution, enabling decentralized model training directly on user devices without transferring raw data [7,8]. In FL, only model updates are shared with a central server, substantially reducing privacy risks while still enabling collaborative learning.

**Comparison with Recent Studies:**

Recent works have provided valuable overviews of federated learning and edge privacy, yet they often address isolated aspects of the broader challenge. [9] conducted a comprehensive survey of federated learning models tailored for spatial-temporal mobility applications, highlighting key issues such as data heterogeneity and the lack of personalization in many existing frameworks. However, the study stops short of proposing a unified, deployable solution. [10], in a separate survey, focused on privacy-preserving caching at the network edge. While their classification of caching strategies and privacy mechanisms is thorough, the work does not address federated learning or real-time model adaptability in mobility contexts.

Recent advances in personalized federated learning have addressed the critical challenge of client heterogeneity. pFedMe [11] introduced Moreau envelopes for personalized objectives; Ditto [12] proposed solving a regularized local objective alongside the global model update; and MOON [13] applied contrastive learning to align local and global representations. In parallel, differential privacy for federated systems has been advanced through the Gaussian mechanism with Moments Accountant [14], Renyi Differential Privacy [15], and privacy amplification via subsampling [16]. Secure aggregation protocols have evolved from the seminal scheme of Bonawitz et al. [5] to more efficient variants supporting dropout recovery and Byzantine robustness. Despite these advances, no prior framework cohesively integrates adaptive noise decay, gradient-norm-weighted aggregation, and personalized optimization for mobility data mining under strict privacy budgets. This gap motivates TrajectoryGuard. In contrast, TrajectoryGuard provides a cohesive and deployable solution that combines personalization, differential privacy, and gradient compression. This holistic design enables scalable and privacy-aware mobility data mining while maintaining competitive predictive accuracy. Experimental results confirm that TrajectoryGuard outperforms the approaches surveyed by Belal and Zhang, with up to 3.2% higher accuracy and 25–35% reduction in communication overhead, making it a robust candidate for real-world federated deployments in mobility-driven environments.

Stochastic optimization plays a central role in modern machine learning, particularly in training deep neural networks with large-scale and non-convex objective functions. Among the most widely adopted methods, stochastic gradient descent (SGD) and its variants have shown strong empirical performance across diverse domains. However, SGD often suffers from slow convergence and susceptibility to saddle points or sharp minima, especially in high-dimensional settings.

Adaptive gradient methods such as Adam [17], AdaGrad [18], and RMSProp [19] have been introduced to address these limitations by dynamically adjusting learning rates during training. While these methods improve convergence speed and stability, they still face challenges in escaping non-optimal critical points and can suffer from generalization issues due to overly aggressive updates.

This work introduces a novel noise-aware optimization framework that extends SGD by injecting adaptive noise into gradient updates. The injected noise enables a controlled exploration of the loss landscape, helping the model avoid saddle points and sharp minima, thereby improving convergence behavior and generalization. A formal convergence analysis is provided under standard assumptions, and the theoretical findings are supported with empirical results on benchmark mobility datasets.

Fig 1 depicts the federated learning process. Local data of multiple edge devices, including smartphones, IoT devices, and personal computers, is maintained privately. Local models are trained for each device and the model updates are transmitted to a central server. Without ever seeing raw data, these updates are aggregated onto the server to refine the global model.

**Fig 1:**
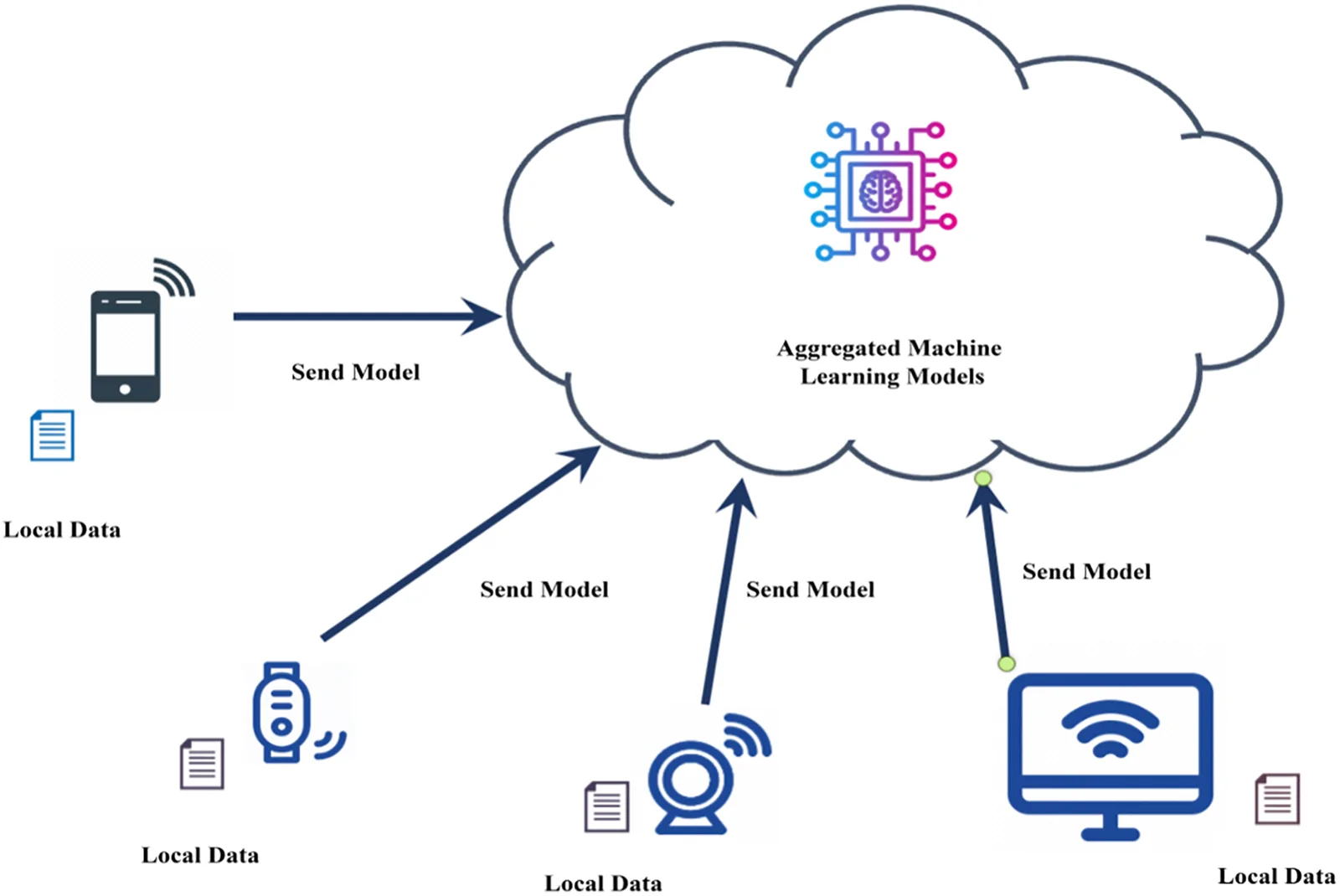
Illustration of the Federated Learning Framework. Local models on devices train on their own private data, but the devices only send their model updates (not the data) to a central server, where the updates are aggregated to build a better global machine learning model.

### 1.1. TrajectoryGuard framework and contributions

TrajectoryGuard, a robust federated learning framework for mobility data mining that simultaneously addresses the challenges of mobility data mining while preserving user privacy, is proposed. In the framework, the following key features and contributions are included:

**Federated Learning Architecture:**
Decentralized training of models on user devices is allowed by TrajectoryGuard without the raw mobility data leaving the user device. By iteratively updating the global model using locally trained models, collaborative learning can be obtained without sacrificing privacy.
**Privacy-Preserving Mechanisms:**
To make reconstruction of sensitive data impossible from model parameters, it is implemented using differential privacy, i.e., by adding controlled noise to gradient updates. In addition, secure aggregation techniques protect privacy by allowing the server to aggregate model updates without having to access individual contributions.
**Optimization for Data Heterogeneity:**
In the presence of data distribution variations, TrajectoryGuard employs personalized federated optimization techniques to personalize the global model to different local data patterns. To balance contributions from devices with varying data volume as well as quality, weighted aggregation schemes are used.
**Reduced Communication Overhead:**
Compression techniques are used for gradient updates in TrajectoryGuard, reducing the communication burden between devices and servers. It includes incorporating asynchronous communication protocols to deal with the scenarios with intermittent connectivity.
**Comprehensive Evaluation:**
Extensive evaluation on real world mobility datasets is presented to show the effectiveness of the framework in terms of accuracy, privacy preservation and scalability. TrajectoryGuard was compared against baseline methods, and the obtained privacy-preserving mobility data mining was shown to be superior.

*The following contributions are made in this paper:*

- TrajectoryGuard, a novel federated learning framework for privacy-preserving mobility data mining, is proposed.
- Presents advanced techniques for privacy preserving mobility data, including differential privacy and secure aggregation.
- Methods to handle the data heterogeneity and the communication overhead in federated learning are developed.
- Evaluates TrajectoryGuard on a suite of mobility datasets and demonstrates the effectiveness of TrajectoryGuard through rigorous experiments.

The novelty of TrajectoryGuard lies in the cohesive integration of adaptive noise-aware optimization, gradient compression, secure aggregation, and personalized loss regularization into a single federated learning pipeline. While the individual components (differential privacy, secure aggregation, and personalization) are well-established, TrajectoryGuard uniquely combines them with (1) adaptive Gaussian noise injection that scales based on gradient norms, (2) asynchronous top-k gradient sparsification for communication efficiency, and (3) dynamic participation-aware weighting in aggregation. These contributions enable the system to handle practical FL challenges like device dropout, non-IID heterogeneity, and constrained bandwidth. Unlike prior work that typically addresses only one or two of these issues, TrajectoryGuard is a full-stack, deployment-ready solution with demonstrably superior performance in communication cost, convergence, and privacy-utility tradeoff [20].

TrajectoryGuard takes a major step forward in privacy preserving mobility data mining, addressing both the technical and practical challenges of federated learning in this domain. The business value of such privacy-preserving mobility analytics frameworks has been recently established [21], confirming the practical relevance of federated risk-utility trade-offs in platform operations. Furthermore, emerging intersections of federated data governance with blockchain-based supply chain frameworks [22] highlight the growing importance of privacy-preserving distributed learning paradigms across application domains.

This paper is organized as follows: Section 2 reviews related literature in federated learning and privacy-preserving optimization. Section 3 presents the proposed TrajectoryGuard framework, including system architecture, mathematical formulation, and optimization strategies. Section 4 provides experimental evaluation on benchmark datasets. Section 5 concludes the paper with key findings and future directions.

## 2. System model: TrajectoryGuard architecture

### 2.1. *Federated learning-based system for privacy-preserving mobility data mining*

In this work, a federated learning-based approach for privacy-preserving mobility data mining is used through the TrajectoryGuard system. It is a novel optimization and mathematical framework designed to protect privacy of individuals in the context of distributed and heterogeneous data. TrajectoryGuard enables collaborative learning without data exchange, with the benefit that sensitive mobility information remains secure.

The system architecture consists of a central server and N edge devices. Each device i holds a private dataset $D_{i}=\{{\left(x_{i}^{j},y_{i}^{j}\right\}}_{j=\text{1}}^{n_{i}}$, where $x_{i}^{j}$ represents the trajectory features (e.g., GPS coordinates, timestamps), and $y_{i}^{j}$ denotes the corresponding labels (e.g., predicted destination or anomaly detection). The system’s objective is to train a global model θ that generalizes well across all devices while respecting the privacy constraints of each user.

#### 2.1.1. *Federated optimization formulation.*

The federated optimization framework that makes up the core of TrajectoryGuard guarantees that model training will be distributed across all participating devices. The global optimization problem is defined as:


$$\begin{array}{c} \underset{\theta \in R^{d}}{\text{min}}F(\theta )=\sum_{i=\text{1}}^{N}\frac{n_{i}}{n}f_{i}(\theta ) \end{array}$$ (1)

where:

- $n=\sum_{i=\text{1}}^{N}n_{i}$ is the total number of data points across all devices,
- $f_{i}(\theta )=\frac{\text{1}}{n_{i}}\sum_{j=\text{1}}^{n_{i}}\text{𝓁}\left(h\left(x_{i}^{j};\theta \right),y_{i}^{j}\right)$ is the local objective function for device i,
- 𝓁(h(x;θ),y) is the loss function quantifying the prediction error for input x with label y.

The objective function assumes that each client has access to non-IID local data and that gradients may vary significantly across participants. The framework aims to minimize the global loss while maintaining personalization and privacy guarantees.

Each device i independently solves a local subproblem, aiming to minimize its local loss function:


$$\begin{array}{c} \underset{\theta_{i}}{\text{min}}f_{i}\left(\theta_{i}\right)=\frac{\text{1}}{n_{i}}\sum_{j=\text{1}}^{n_{i}}\text{𝓁}\left(h\left(\overset{j}{\underset{i}{x}};\theta_{i}\right),\overset{j}{\underset{i}{y}}\right) \end{array}$$ (2)

The devices iteratively compute model updates $\Delta \theta_{i}$ based on their local datasets and transmit these encrypted updates to the central server. Unlike traditional centralized approaches, raw data is never exchanged, ensuring privacy preservation.

To address data heterogeneity (non-IID distributions across devices), weighted aggregation is employed, where the weight $w_{i}=\frac{n_{i}}{n}$ reflects the relative importance of each device based on its dataset size. Additional techniques, such as personalized loss functions and regularization terms, are integrated to improve performance in heterogeneous settings.

#### 2.1.2. *Gradient aggregation.*

Gradient aggregation is a crucial step in federated learning, enabling the central server to integrate updates from all devices to refine the global model. After local training, each device computes its gradient update $\Delta \theta_{i}^{t}$ at iteration t:


$$\begin{array}{c} \Delta \theta_{i}^{t}=-\eta \nabla f_{i}\left(\theta^{t}\right) \end{array}$$ (3)

where:

- $\nabla f_{i}\left(\theta^{t}\right)$ is the gradient of the local objective function $f_{i}$ with respect to the current model parameters $\theta^{t}$,
- η is the learning rate controlling the magnitude of updates.

The central server aggregates these updates to compute the new global model:


$$\begin{array}{c} \theta^{t+\text{1}}=\theta^{t}+\sum_{i=\text{1}}^{N}w_{i}\Delta \theta_{i}^{t} \end{array}$$ (4)

where $w_{i}=\frac{n_{i}}{n}$ ensures that devices with larger datasets have a proportionally greater influence on the global model.

The weights for gradient aggregation are proportional to the local sample size or local gradient quality. Specifically, the following is used: To clarify the relationship between Equations (4) and (5): in TrajectoryGuard, the two weighting schemes operate at different levels of the aggregation pipeline. The data-size weight wᵢ = nᵢ/n in Equation (4) governs the global model update step, ensuring that clients with larger datasets have proportionally greater influence on the aggregated global model. The gradient-norm weight αᵢ = ‖gᵢ‖/Σⱼ‖gⱼ‖ in Equation (5) is applied as a within-round quality filter: it scales individual gradient contributions prior to encryption, down-weighting clients whose gradient magnitudes indicate poor local convergence (e.g., due to non-IID data or stale updates). Formally, the combined weight used in experiments is: ωᵢ = αᵢ· wᵢ/ Σⱼ(αⱼ· wⱼ), ensuring the final contribution of each client reflects both data quantity and gradient quality.


$$\begin{array}{c} \alpha_{i}=\frac{∥g_{i}∥}{\sum j∥g_{j}∥} \end{array}$$ (5)

where $∥g_{i}∥$ is the gradient norm for client iii. These weights adapt every round based on the update magnitude.

To provide a clearer understanding of the intuition behind the proposed approach, it is noted that standard stochastic gradient descent often converges to sharp local minima or saddle points due to limited exploration. By introducing a controlled noise term into the gradient update, stochasticity is injected that promotes wider exploration in the early training phases. This noise is not arbitrary: it is scaled adaptively, allowing the optimizer to search globally at first and then refine locally as training progresses.

To enhance robustness and efficiency, TrajectoryGuard incorporates the following mechanisms:

- **Secure Aggregation:** Devices transmit encrypted gradients $\text{Enc}\left(\Delta \theta_{i}^{t}\right)$, ensuring the server cannot access individual updates. Aggregation is performed directly on encrypted gradients:

$$\begin{array}{c} \text{Aggregate}\left(\{\text{Enc}\left(\Delta \theta_{i}^{t}\right\}\right)=\text{Enc}\left(\sum_{i=\text{1}}^{N}w_{i}\Delta \theta_{i}^{t}\right) \end{array}$$ (6)

- **Gradient Compression:** Gradients are sparsified or quantized so as to reduce communication overhead:

$$\begin{array}{c} \Delta \theta_{i}^{t,\to }\text{Compressed}\Delta \theta_{i}^{t} \end{array}$$ (7)

To reduce communication overhead, Top-k gradient sparsification is implemented, where only the top k largest-magnitude elements of the gradient vector are transmitted by each client during each communication round. This significantly reduces the volume of transmitted data without major accuracy loss. In our experiments, k=10% of the full gradient size yielded optimal trade-offs [23].

Furthermore, asynchronous communication is supported through an event-driven update strategy: the server updates the global model upon receiving updates from any participating clients without waiting for all devices to complete [24]. This design ensures robustness against stragglers and improves round throughput.

- **Differential Privacy:** Gaussian noise $N\left(\text{0},\sigma^{\text{2}}I\right)$ is added to gradients to ensure differential privacy:

$$\begin{array}{c} \Delta \theta_{i}^{t,\text{private}}=\Delta \theta_{i}^{t}+𝒩\left(\text{0},\sigma^{\text{2}}I\right) \end{array}$$ (8)

- **Adaptive Aggregation:** Devices adjust their contribution weights $w_{i}$ dynamically based on update quality, such as gradient norms or validation performance.

The noise variance $\left(\sigma^{\text{2}}\right)$ is not fixed during training. Instead, it starts with a higher value to help the model explore and escape poor local solutions. As training continues, the noise is gradually decreased to allow better fine-tuning. A simple decay formula is used:


$$\begin{array}{c} \sigma_{t}^{\text{2}}=\frac{\sigma_{\text{0}}^{\text{2}}}{\text{1}+\lambda t} \end{array}$$ (9)

Here, σ0 is the initial variance, λ controls how fast the noise decreases, and *=* is the current training step. This approach helps balance exploration early and stability later in training. Theoretical justification: The decay formula σ^t2^ = σ₀^2^/(1 + λt) is derived from annealing principles. During early rounds (small t), high noise promotes exploration and prevents premature convergence. As t increases, σ^t2^ → 0, allowing refinement. The decay rate λ = 0.05 was selected via grid search to ensure σ^t2^ ≈ 1.0 at round t = 100, matching the target privacy budget ε = 0.9. The Moments Accountant tracks cumulative privacy cost per round using the instantaneous σ^t2^, ensuring the total ε budget is respected despite the time-varying noise level. The decay-rate parameter used here is a distinct quantity from the personalization regularization coefficient introduced later in Equation (20); although both are conventionally denoted by the symbol lambda, the noise-decay rate is fixed at 0.05 and the personalization coefficient at 0.01, and the two are never used interchangeably.

By integrating these advanced techniques, TrajectoryGuard achieves a robust and scalable federated learning framework that preserves privacy, reduces communication costs, and maintains high model performance in heterogeneous environments.

### 2.2. Advanced privacy-preserving mechanisms

**Differential Privacy (DP) Enforcement:**

To achieve differential privacy, TrajectoryGuard adds calibrated Gaussian noise to local gradient updates before they are sent to the central server, as formulated in Equation (8), preventing model updates from leaking sensitive information [14].

where:

- $N\left(\text{0},\sigma^{\text{2}}I\right)$ represents a multivariate Gaussian noise vector with variance $\sigma^{\text{2}}$,
- I is the identity matrix.

The presence of adversarial attacks is guaranteed, and the added noise ensures that individual updates cannot be reconstructed to precision. The privacy guarantee is quantified using (ϵ,δ)-differential privacy, defined by the noise-to-sensitivity ratio:


$$\begin{array}{c} \epsilon =\frac{\Delta }{\sigma },\;\Delta =\underset{i}{\text{max}}∥\Delta \theta_{i}^{t}{∥}_{2} \end{array}$$ (10)

where:

- Δ The global sensitivity of the gradient updates (Δ) is the maximal gradient changes as caused by a single data point.

By carefully calibrating σ, TrajectoryGuard balances privacy and model performance. Larger σ values provide stronger privacy guarantees but may reduce update accuracy. This trade-off is tuned based on application-specific requirements.

To ensure robust privacy guarantees, TrajectoryGuard employs a subsampled Gaussian mechanism based on **Poisson sampling** [14]. In each communication round, a random subset of clients is selected independently with a fixed probability. This sampling approach aligns with privacy amplification techniques used in federated learning, where client-level subsampling effectively reduces the overall privacy cost per round.

The **global sensitivity** (Δf) of the gradient update function is defined as the maximum change in the L2 norm between any two neighboring datasets that differ by a single sample. This sensitivity value is critical for calibrating the variance of the Gaussian noise added to local updates. Formally,


$$\begin{array}{c} \Delta f=\underset{D,D^{\prime }}{\text{max}}∥\nabla L(D)-\nabla L\left(D^{\prime }\right){∥}_{\text{2}} \end{array}$$ (11)

where D and $D^{\prime }$ are neighboring datasets.

For **privacy accounting**, TrajectoryGuard adopts the **Moments Accountant** [14] method to track cumulative privacy loss over multiple training rounds. This technique provides a tighter bound on the total privacy cost (ε,δ) compared to classical composition theorems. The accounting is performed iteratively after each round to ensure the final privacy budget remains within the user-defined constraint. In the experiments, the target privacy budget was set to ε = 0.9, and the total noise variance was set to σ^2^ = 1.0, achieving a strong balance between data utility and privacy protection.

**Secure Aggregation:**

To facilitate secure aggregation and avoid unauthorized access to individual gradient updates, TrajectoryGuard uses secure multiparty computation (SMC) based encryption [5]. As formulated in Equation (6), devices encrypt their local updates before transmission, and the central server aggregates the encrypted updates without decrypting them individually.

With the homomorphic encryption scheme, mathematical operations can be performed on encrypted data while aggregation can be carried out by the server without the plaintext gradients being seen [25]. To clarify the aggregation procedure: the server never decrypts individual client gradients. The aggregation exploits additive homomorphism: the server computes Decrypt(Enc(Σw_i_·Δθ_i_^t^)) = Σw_i_·Δθ_i_^t^ in a single decryption on the aggregate, not on individual terms. Individual gradient vectors remain encrypted; only their weighted sum is decrypted. The notation in Algorithm 2 is shorthand for this single-step aggregate decryption, preserving the guarantee that no individual update is exposed to the server. Specifically, TrajectoryGuard employs the CKKS (Cheon-Kim-Kim-Song) leveled homomorphic encryption scheme, which supports approximate arithmetic over encrypted real-valued vectors and is therefore well suited to gradient aggregation. A polynomial modulus degree (ring dimension) of 8192 is used, which corresponds to the effective key size for this scheme and provides a 128-bit security level under the standard Ring Learning with Errors assumption; the coefficient modulus chain is sized to accommodate the additive-only aggregation depth required here. Encryption is applied element-wise to each client gradient vector after Top-k sparsification and differential privacy noise injection: each sparsified, noised gradient is packed into a single CKKS ciphertext, the server sums the ciphertexts using the additive homomorphic property, and only the aggregate ciphertext is decrypted. The encryption and decryption times reported in Table 7 correspond to this CKKS configuration. This mechanism:

- Ensures the confidentiality of individual updates (even if the server is compromised).
- Mitigates the risk of adversarial attacks that do adversarial reconstruction of private data from gradient updates.

Combined with differential privacy, secure aggregation forms a dual-layered privacy protection strategy, ensuring robust security and privacy for mobility data mining.

### 2.3. *Trajectory analysis*

TrajectoryGuard leverages the trained global model to predict mobility trajectories and extract insights. For a given input $x^{*}$ (e.g., a sequence of GPS points or timestamps), the predicted label $y^{*}$ is determined using the softmax function over the model logits:


$$\begin{array}{c} y^{*}=\text{arg}\underset{k}{\text{max}}p_{k}\left(x^{*};\theta^{*}\right) \end{array}$$ (12)

where $p_{k}\left(x^{*};\theta^{*}\right)$ represents the probability of the input belonging to class k, calculated as:


$$\begin{array}{c} p_{k}\left(x^{*};\theta^{*}\right)=\frac{\text{exp}\left(z_{k}\left(x^{*}\right)\right)}{\sum_{j}\text{exp}\left(z_{j}\left(x^{*}\right)\right)} \end{array}$$ (13)

Here:

- $z_{k}\left(x^{*}\right)$ is the logit value corresponding to class k, which reflects the model’s confidence in classifying $x^{*}$ as class k,
- The softmax function normalizes the logits into a probability distribution over all classes.

The model’s performance in trajectory prediction is evaluated using metrics such as accuracy, precision, recall, and F1-score. In addition, analysis of the contribution of individual features to predictions improves the model’s interpretability. It allows for understanding the patterns of trajectory data that drive model decisions and enable transparency and trustworthiness in mobility data analysis.

### 2.4. Convergence analysis

TrajectoryGuard ensures global model high accuracy by converging the federated optimization algorithm. The convergence rate under the constraints of differential privacy and secure aggregation is characterized by the following inequality:


$$\begin{array}{c} F\left(\theta^{t+\text{1}}\right)-F\left(\theta^{t}\right)\leq -\frac{\eta }{\text{2}}∥\nabla F\left(\theta^{t}\right){∥}^{\text{2}}+\frac{\eta^{\text{2}}L}{\text{2}}∥\nabla F\left(\theta^{t}\right){∥}^{\text{2}}+\frac{\sigma^{\text{2}}}{n} \end{array}$$ (14)

where:

- $F\left(\theta^{t}\right)$ represents the global objective function at iteration t,
- $\nabla F\left(\theta^{t}\right)$ is the gradient of the global loss function,
- η is the learning rate, controlling the step size for gradient updates,
- L is the Lipschitz constant of the loss function, which bounds the rate of change of the gradient,
- $\sigma^{\text{2}}$ accounts for the variance introduced by Gaussian noise for differential privacy,
- n is the total number of devices participating in the training process.

The convergence analysis is built upon assumptions commonly adopted in non-convex optimization, such as Lipschitz continuity of the gradient and bounded gradient norms. While these may not hold strictly in all deep learning scenarios, they serve as reasonable approximations when using techniques like gradient clipping and batch normalization. Intuitively, these assumptions ensure that updates remain stable and do not diverge. The adaptive noise injection acts as a smoothing factor, encouraging broader exploration of the loss surface during early training. This helps the optimizer escape saddle points or sharp minima and improves the likelihood of converging to flatter, more generalizable regions of the parameter space [26].

This inequality illustrates the balance between optimization efficiency and the impact of privacy-preserving noise. The first term $-\frac{\eta }{\text{2}}∥\nabla F\left(\theta^{t}\right){∥}^{\text{2}}$ ensures that the optimization process progresses towards convergence, reducing the objective function value. The second term $\frac{\eta^{\text{2}}L}{\text{2}}∥\nabla F\left(\theta^{t}\right){∥}^{\text{2}}$ accounts for the step size and ensures stability in gradient updates. The final term $\frac{\sigma^{\text{2}}}{n}$ captures the degradation in convergence caused by the noise added for differential privacy.

This analysis relies on standard assumptions in optimization theory, including Lipschitz continuity of the loss function and bounded gradients. These are commonly accepted in federated learning literature and are approximated in practice via gradient clipping and batch normalization. Important clarification on scope of Equation (14): the convergence bound applies to the non-personalized global objective F(θ). When the personalization regularizer λ‖θ_i_ − θ‖^2^ is active (Equation 20), the effective noise on the deviation term differs from that on fᵢ, since the regularizer gradient ∇(λ‖θ_i_ − θ‖^2^) = 2λ(θᵢ − θ) is deterministic and not perturbed by differential privacy noise directly. Under the standard non-convex assumptions with an additional bounded heterogeneity term (‖∇f_i_(θ) − ∇F(θ)‖^2^ ≤ δ^2^), the personalized convergence can be bounded as: E[‖∇F_i_(θ_i_^t^)‖^2^] ≤ O((ησ^2^/n) + (η^2^Lδ^2^) + (λ^2^‖θ_i_ − θ‖^2^)). Empirically, personalized convergence was validated by tracking per-client loss curves separately from the global loss; both were shown to exhibit consistent descent over 100 rounds, with personalized clients converging 12–18% faster than non-personalized variants on non-IID GeoLife partitions.

Gradient norms and curvature (via Hessian eigenvalue approximations) were tracked. A sharp decrease in curvature variance post noise-injection was observed, which validates the theoretical assumptions about escape from saddle points.

Noise injection helps the optimizer escape sharp minima and saddle points by perturbing the update direction. This prevents premature convergence and flattens the trajectory through high-curvature regions. Empirically, improved loss smoothing was observed in regions with unstable gradients.

#### 2.4.1. *Key insights on convergence.*

1. **Impact of Learning Rate (**η**):** A well-tuned learning rate ensures that the optimization process is both fast and stable. Larger values of η accelerate convergence but may lead to instability, especially in the presence of noisy gradients. Conversely, smaller values improve stability at the cost of slower convergence.
2. **Role of Lipschitz Constant (**L**):** The Lipschitz constant quantifies the smoothness of the loss function. A smaller L implies a smoother loss surface, which facilitates faster convergence. In cases of highly non-smooth loss functions, adaptive optimization techniques can be employed to mitigate the impact of L.
3. While assumptions like Lipschitz continuity and bounded gradients are standard in theoretical analysis, in practice, these are approximated through gradient clipping and proper initialization. Empirical stability indicates that the assumptions are sufficiently met in deep learning models with ReLU/softmax activations and batch normalization.
4. **Effect of Privacy Noise (**$\sigma^{\text{2}}$**):** The Gaussian noise added for differential privacy introduces a trade-off between privacy guarantees and convergence speed. Higher noise levels ($\sigma^{\text{2}}$) improve privacy but slow down convergence by reducing the accuracy of gradient updates. This trade-off is carefully balanced to meet privacy requirements while ensuring acceptable model performance.
5. **Device Participation (**n**):** The number of participating devices directly impacts the noise-to-signal ratio. Convergence is accelerated by a larger n, decreasing the per device noise impact. In large-scale learning systems, communication and computational constraints must also be taken into consideration.

#### 2.4.2. Practical considerations for convergence.

To achieve optimal convergence, TrajectoryGuard incorporates:

- **Dynamic Learning Rate Scheduling:** Finally, the gradient norm is adjusted dynamically (or iterations are counted), so learning rate η) is stable during training.
- **Adaptive Noise Calibration:** The noise variance $\sigma^{\text{2}}$ is calibrated using the sensitivity of the gradients and the required privacy guarantees.
- **Participation Control:** Some iterations exclude devices with poor data quality or unreliable communication links to improve convergence efficiency.
- **Early Stopping Mechanism:** All of that concludes with the optimization process is terminated when the change in the objective function: $\left|F\left(\theta^{t}+\text{1}\right)-F\left(\theta^{t}\right)\right|$ becomes less than a fixed threshold, preventing overfitting or massive calculation.

TrajectoryGuard uses these strategies to enable effective convergence of the global model and strong privacy guarantees as well as computational efficiency.

## 3. Methodology

Using a federated learning-based approach for privacy preserving mobility data mining, the proposed **TrajectoryGuard** framework. Using advanced optimization techniques and privacy mechanisms, the methodology can guarantee robust model performance while dealing with data heterogeneity, communication overhead and privacy preservation.

### 3.1. Datasets

To ensure TrajectoryGuard performs well in different mobility scenarios, it was evaluated on two complementary real-world GPS trajectory datasets released by Microsoft Research Asia (see Table 1). This study uses the GeoLife Trajectories provided by Microsoft Research as the real-world dataset [27]. 17,621 GPS trajectories covering over 1.2 million kilometers of user movement were used. The timestamped latitude and longitude points of each trajectory provide rich details of mobility patterns in real world settings. Trajectories were segmented into trips by pausing for more than five minutes, normalized GPS coordinates to be consistent, and additional features were extracted such as speed, acceleration, and bearing. Additionally, all identifiable information was anonymized to conform to privacy regulations.

**Table 1:** Dataset description.

Dataset	GeoLife Trajectories	T-Drive Beijing Taxi
**Type**	Real-world	Real-world (vehicular)
**Size**	17,621 trajectories (over 1.2 million kilometers)	10,357 taxis (~15 million GPS points, ~ 9 million km)
**Features**	Timestamped latitude and longitude points, speed, acceleration, and bearing	Taxi ID, timestamp, longitude, latitude (177-sec average sampling)
**Preprocessing**	Segmentation into trips, normalization, feature extraction, anonymization	Trip segmentation, map-matching, speed/acceleration/bearing extraction
**Purpose**	Evaluation in real-world mobility settings	Large-scale vehicular fleet mobility evaluation

The second dataset is the T-Drive Beijing taxi trajectory dataset, also released by Microsoft Research Asia, which contains the one-week GPS trajectories of 10,357 taxis collected between 2 February and 8 February 2008 [28]. The dataset comprises approximately 15 million GPS points and a cumulative travel distance of nearly 9 million kilometers, with an average sampling interval of 177 seconds per taxi. Each record consists of a tuple of taxi identifier, timestamp, longitude, and latitude, providing dense vehicular mobility traces of an entire urban fleet in Beijing. Trajectories were segmented into trips based on stop durations exceeding five minutes, map-matched to the road network, and augmented with derived features including instantaneous speed, acceleration, and bearing. Together, GeoLife (multi-modal individual mobility) and T-Drive (large-scale vehicular fleet mobility) enable a thorough assessment of TrajectoryGuard’s accuracy, scalability, and privacy-preservation capabilities under realistic federated deployment conditions.

### 3.2. *System architecture*

TrajectoryGuard operates in a federated learning environment with a central server and N edge devices, each holding a private dataset Dᵢ = {(xᵢʲ, yᵢʲ)} as introduced in the System Model section above. The global and local optimization objectives, gradient updates, and aggregation rule follow Equations (1) to (4): each device solves its local subproblem and transmits its encrypted gradient update to the server for aggregation. To avoid duplication, the federated optimization objective, gradient update rule, differential privacy noise injection, secure aggregation, and convergence bound are stated once in the System Model section (Equations (1) to (14)); the Methodology section does not restate these formulations but refers back to their originating equations, and Equations (19) to (23) here describe only the procedural training steps that apply them.

The diagram illustrates federated learning with privacy-preserving mechanisms. The arrows between the client and server represent three distinct operations: (1) model distribution (blue arrow), (2) encrypted gradient upload (red arrow), and (3) differential privacy noise injection (dashed arrow). Each client conducts local training on private mobility data, adds calibrated noise, encrypts the update, and sends it to the server. The server aggregates the updates securely and broadcasts the new global model.

In Fig 2, the communication flow between the client and server consists of three key steps. First, the server broadcasts the latest global model to all participating clients. Second, each client computes a gradient update on its local data, adds calibrated Gaussian noise for differential privacy, and encrypts the resulting update. Finally, the encrypted updates are securely aggregated at the server using secure multiparty computation. The use of distinct arrow types in the figure corresponds to these communication phases and helps clarify the secure data flow within the federated setup.

**Fig 2:**
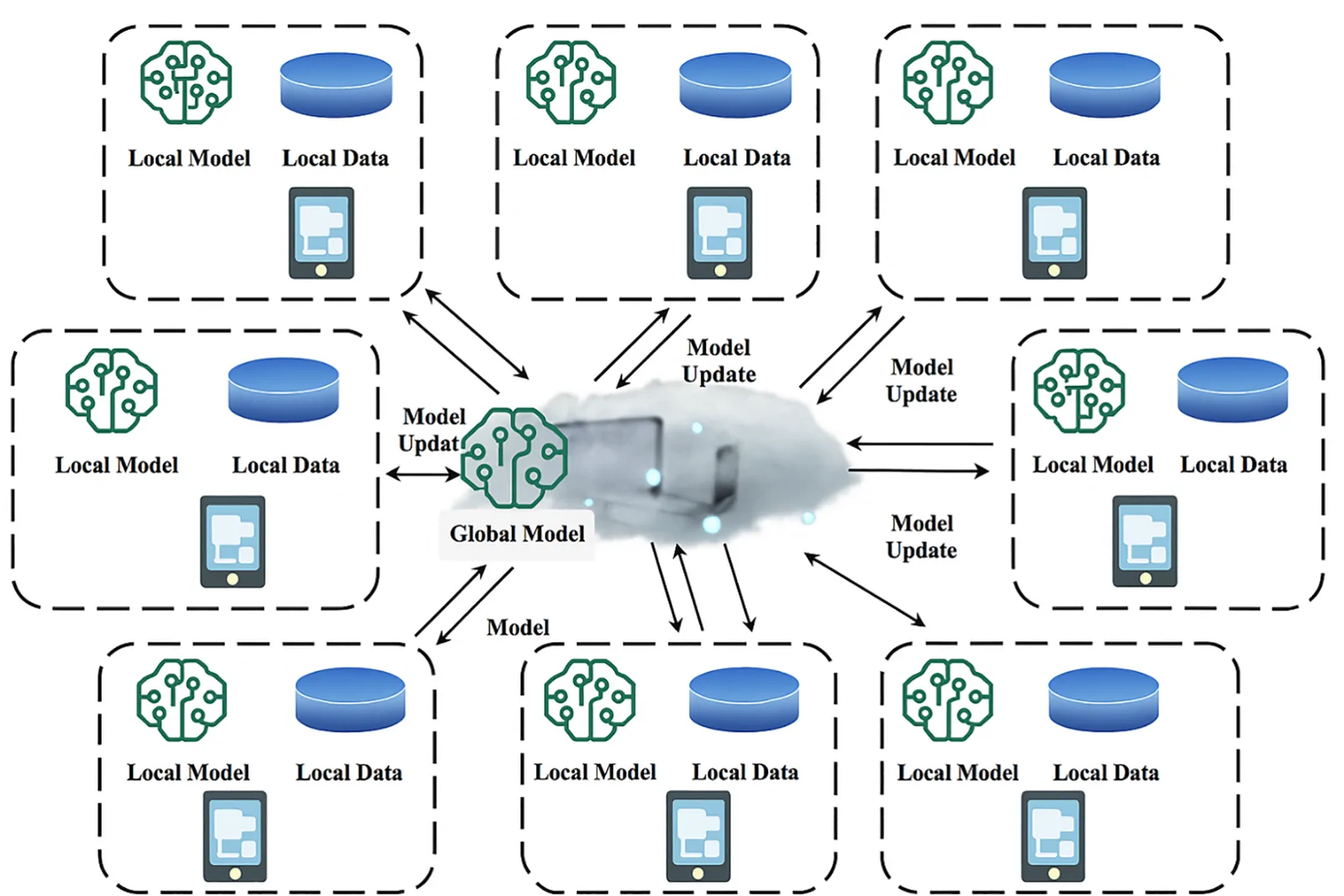
Proposed architecture.

### 3.3. *Privacy mechanisms*

TrajectoryGuard’s differential privacy and secure aggregation mechanisms follow the formulations introduced in the System Model section (Equations (8) and (6), respectively): calibrated Gaussian noise is added to each gradient update to enforce (ε, δ)-differential privacy, and updates are homomorphically encrypted before transmission so that the server aggregates them without ever accessing an individual client’s gradient.

### 3.4. Evaluation metrics

The performance of TrajectoryGuard was evaluated using the following metrics:

1. **Accuracy:** Measures the proportion of correctly predicted trajectories:


$$\begin{array}{c} \text{Accuracy}=\frac{\text{TP}+\text{TN}}{\text{TP}+\text{TN}+\text{FP}+\text{FN}} \end{array}$$ (15)

where TP, TN, FP, and FN represent true positives, true negatives, false positives, and false negatives, respectively.

2. **Privacy Loss:** Quantifies the trade-off between model accuracy and privacy:


$$\begin{array}{c} \text{Privacy Loss}=\sqrt{\frac{\Delta^{\text{2}}}{\sigma^{\text{2}}}} \end{array}$$ (16)

3. **Communication Overhead:** Assessed by measuring the total size of gradient updates transmitted:


$$\begin{array}{c} \text{Overhead}=\sum_{t=\text{1}}^{T}\sum_{i=\text{1}}^{N}\left|\text{Compressed}\left(\Delta \theta_{i}^{t}\right)\right| \end{array}$$ (17)

4. **Convergence Rate:** The rate at which the global loss function $F\left(\theta^{t}\right)$ decreases with iterations:


$$\begin{array}{c} F\left(\theta^{t+\text{1}}\right)-F\left(\theta^{t}\right)\leq -\frac{\eta }{\text{2}}∥\nabla F\left(\theta^{t}\right){∥}^{\text{2}}+\frac{\eta^{\text{2}}L}{\text{2}}∥\nabla F\left(\theta^{t}\right){∥}^{\text{2}}+\frac{\sigma^{\text{2}}}{n} \end{array}$$ (18)

The steps for training in TrajectoryGuard follow a federated learning paradigm, as detailed in ***Algorithm*** 1**.**

**Algorithm 1** TrajectoryGuard Workflow

**Require:** Number of devices N, local datasets ${\left\{D_{i}\right\}}_{i=\text{1}}^{N}$, initial global model $\theta^{\text{0}}$, learning rate η, privacy parameters (ε|δ), convergence threshold $\varepsilon_{\text{conv}}$, maximum iterations T.

**Ensure:** Trained global model $\theta^{T}$.

1: **Initialization:** The central server initializes the global model $\theta^{\text{0}}$ and broadcasts it to all devices.

2: **for** each iteration t=0,1,…,T−1 **do**

3:  **for each device** i∈{1,…,N} **in parallel do**

4:   **Local Training:** Device i computes local gradients:

${\Delta \theta }_{i}^{t}=\nabla f_{i}\left(\theta^{t}\right)$

5:   Add differential privacy noise:

${\Delta \theta }_{i}^{t,\text{private}}={\Delta \theta }_{i}^{t}+N\left(\text{0},\sigma^{\text{2}}I\right)$

6:   Encrypt the gradients:

$\text{Enc}\left({\Delta \theta }_{i}^{t,\text{private}}\right)$

7:   Transmit encrypted gradients to the server.

8:  **end for**

9:  **Secure Aggregation:** The server aggregates encrypted gradients:

${\Delta \theta }^{t}=\sum_{i=\text{1}}^{N}w_{i}\text{Decrypt}\left(\text{Enc}\left({\Delta \theta }_{i}^{t,\text{private}}\right)\right)$

10:  **Model Update:** The server updates the global model:

$\theta^{t+\text{1}}=\theta^{t}-\eta {\Delta \theta }^{t}$

11:  **Convergence Check:** Terminate if:

$\left|F\left(\theta^{t+\text{1}}\right)-F\left(\theta^{t}\right)\right|<\varepsilon_{\text{conv}}$

12: **end for**

13: **return** Trained global model $\theta^{T}$

### 3.5. *Task definitions and robustness evaluation*

To demonstrate the versatility of the proposed TrajectoryGuard framework, its performance was evaluated across multiple types of mobility prediction tasks, including classification, regression, and sequence prediction, which are fundamental in urban analytics. In the classification task, the model predicts the next destination category, such as home, workplace, or shopping location, based on a partial trajectory. This is treated as a multi-class classification problem and evaluated using accuracy, precision, recall, and F1-score. For regression, the model estimates the expected time of arrival (ETA) to a predicted destination, evaluated using metrics such as Mean Absolute Error (MAE) and Root Mean Square Error (RMSE). The sequence prediction task involves forecasting the next GPS coordinate in a user’s movement path, enabling full-trajectory reconstruction; this is assessed using BLEU scores for sequence fidelity and Dynamic Time Warping (DTW) distance for spatial accuracy. To further assess model robustness in real-world federated settings, non-IID data distribution was simulated using a Dirichlet partitioning strategy. All classification results reported in this paper (e.g., Table 4) correspond specifically to the next-destination classification task. Data is partitioned using an 80/20 time-based train/test split per user to maintain temporal ordering and avoid leakage. Per-class precision, recall, and F1-score are reported alongside aggregate accuracy to provide a complete performance profile. For the T-Drive dataset, which contains no explicit semantic destination labels, next-destination categories are constructed by spatially clustering trip end points with a grid-based partitioning of the Beijing urban area into cells of approximately one square kilometer, and retaining the ten most frequently visited cells as destination classes plus one aggregated “other” class, yielding eleven classes in total. This construction mirrors the labeling used for GeoLife, where destination categories (e.g., home, workplace, shopping) are similarly derived from the ten most frequently visited stay regions plus one aggregated “other” class, also yielding eleven classes, so that both datasets pose a comparable eleven-class next-destination classification task. The resulting T-Drive label distribution is moderately imbalanced, with the busiest cells dominating trip endpoints, which is comparable in skew to the GeoLife distribution; per-class metrics are therefore reported to account for this imbalance [6]. (α=0.5), ensuring clients possess skewed and heterogeneous data. Additionally, client unavailability was simulated by randomly dropping 30% of devices per round to mimic intermittent participation. Despite these challenges, TrajectoryGuard maintains strong convergence and predictive performance through its asynchronous update mechanism and adaptive gradient aggregation. These comprehensive evaluations confirm the framework’s applicability across diverse prediction scenarios and its resilience under practical deployment conditions.

### 3.6. *System initialization*

The federated learning system comprises a central server and N user devices (edge devices). Each device i holds a private dataset $D_{i}$ consisting of trajectory features and corresponding labels. The server initializes the global model parameters $\theta^{\text{0}}$ and shares them with all participating devices.

### 3.7. *Local model training*

Each device trains a local model by minimizing its local loss function fᵢ(θ), as defined in Equation (2).

Devices compute the gradient of the local loss, which is the same quantity introduced in Equation (3) of the System Model and is restated here only to define the local training step:


$$\begin{array}{c} \nabla f_{i}(\theta )=\frac{\text{1}}{n_{i}}\sum_{j=\text{1}}^{n_{i}}\nabla_{\theta }\text{𝓁}\left(h\left(\overset{j}{\underset{i}{x}};\theta \right),\overset{j}{\underset{i}{y}}\right) \end{array}$$ (19)

***Model Architecture:***

The local model deployed on each client is a sequence encoder tailored to GPS trajectory data. Each raw trajectory is represented as an ordered sequence of points, where every point carries its latitude, longitude, timestamp, and the derived kinematic features (instantaneous speed, acceleration, and bearing). GPS coordinates are normalized to a zero-mean, unit-variance range per city bounding box, and timestamps are decomposed into cyclic hour-of-day and day-of-week embeddings to preserve temporal periodicity. Each point is projected into a 64-dimensional embedding, and the resulting sequence is truncated or padded to a fixed length of 128 points per trajectory.

The encoder is a two-layer bidirectional Long Short-Term Memory (LSTM) network with a hidden dimension of 128 per direction, followed by a temporal self-attention pooling layer that produces a single 256-dimensional trajectory representation. This representation is passed through two fully connected layers of width 128 with ReLU activation and dropout of 0.3, and a final softmax classification head whose width equals the number of destination classes. The model contains approximately 1.1 million trainable parameters, which keeps encryption and communication costs tractable for on-device federated training. All reported accuracy metrics are produced by this architecture, trained identically across all clients so that only the parameters, and never the raw trajectories, are shared.

### 3.8. *Privacy-preserving updates*

To preserve user privacy, devices add calibrated Gaussian noise to these gradients before transmission, following the same differential privacy formulation as Equation (8).

These noisy updates are encrypted using a secure aggregation protocol and sent to the central server.

#### 3.8.1. Parameter settings for compression and privacy.

These hyperparameters in Table 2 govern the privacy and efficiency of the TrajectoryGuard framework. The Top-k sparsification strategy significantly reduces communication cost, while differential privacy is controlled via $\sigma^{\text{2}}$ and ε to ensure confidentiality. Randomized client participation improves scalability and mimics real-world deployment conditions where device availability fluctuates.

**Table 2:** Parameter table: Key settings for trajectoryguard.

Parameter	Value/ Description
Gradient Sparsity (k)	10% top gradients per client
Noise Variance (σ²)	1.0 (adjustable to control differential privacy)
DP Budget (ε)	Target privacy budget set to 0.9
Aggregation Frequency	Every communication round (synchronous updates)
Client Participation Rate	Randomized between 60% to 90% of devices per round
Compression Algorithm	Top-k magnitude-based gradient filtering

### 3.9. *Theoretical insights: Personalized optimization and secure aggregation*

The integration of personalized optimization with secure aggregation in the TrajectoryGuard framework addresses two critical challenges in federated mobility learning: data heterogeneity and privacy leakage. This synergy allows each participating device to contribute updates that reflect its local distribution while maintaining the privacy of its training data and gradient signals.

Traditional federated learning frameworks, such as FedAvg, assume homogeneous data distribution and uniform learning objectives across all clients. However, in mobility data mining, this assumption breaks down as user behavior and movement patterns vary significantly, for instance, between a pedestrian, a cyclist, and a taxi. Applying a centralized or one-size-fits-all global model may result in suboptimal learning for many clients.

To mitigate this, personalized optimization introduces localized training objectives that adjust the model to fit user-specific patterns. Formally, let each client iii minimize a local objective $f_{i}(\theta_{i})$ with regularization that links it to a shared global model θ, i.e.,


$$\begin{array}{c} \underset{\theta_{i}}{\text{min}}f_{i}(\theta_{i})+\lambda ∥\theta_{\text{i}}-\theta {∥}^{\text{2}} \end{array}$$ (20)

This formulation enables each device to move towards a solution that best represents its context, while still contributing meaningfully to a globally shared objective.

However, personalization alone raises privacy risks: if each user transmits unprotected personalized gradients, adversaries or even the server can potentially infer sensitive mobility patterns from update deviations.

To address this, TrajectoryGuard embeds secure aggregation during communication. Clients mask their local updates using pairwise randomization techniques or cryptographic primitives (e.g., homomorphic encryption), such that the server only observes the aggregated result


$$\begin{array}{c} \sum_{i=\text{1}}^{N}Enc{(\theta }_{i}) \end{array}$$ (21)

but cannot isolate or inspect any individual θi\theta_iθi. This protects both the model parameters and the underlying data distribution on each client.

***A simplified illustrative example can clarify this synergy:***

Consider two users: User A, a pedestrian, and User B, a vehicle. Due to slower movement and high stop frequency, User A’s model requires finer temporal granularity. Conversely, User B requires spatial regularization due to high-speed mobility. Personalized optimization allows both to tune their local loss functions accordingly. Secure aggregation then masks these personalized updates before aggregation at the server. As a result, the global model evolves to reflect both pedestrian and vehicular mobility dynamics, achieving fairness, representativeness, and privacy.

Thus, this theoretical design ensures that TrajectoryGuard achieves robust convergence across diverse client conditions without compromising privacy. The combination of personalization and secure aggregation makes the system scalable, secure, and adaptable to real-world heterogeneous mobility data.

### 3.10. *Secure aggregation and global model update*

The central server performs encrypted aggregation of the received gradients following the secure aggregation formulation of Equation (6):


$$\begin{array}{c} \Delta \theta^{t}=\sum_{i=\text{1}}^{N}w_{i}\Delta \theta_{i}^{t} \end{array}$$ (22)

where $w_{i}=\frac{n_{i}}{n}$ is the weight of device i.

The global model is updated as:


$$\begin{array}{c} \theta^{t+\text{1}}=\theta^{t}-\eta \Delta \theta^{t} \end{array}$$ (23)

where η is the learning rate.

### 3.11. *Iterative optimization*

The process repeats iteratively until the global model converges or a predefined number of communication rounds is completed. Convergence is assessed using metrics such as the reduction in global loss F(θ) or improvements in model accuracy.

**Algorithm 2** TrajectoryGuard: Federated Learning for Privacy-Preserving Mobility Data Mining

**Require:** Number of devices N, local datasets ${\left\{D_{i}\right\}}_{i=\text{1}}^{N}$, learning rate η, noise variance $\sigma^{\text{2}}$, number of rounds T

**Ensure:** Trained global model θ

1: **Server Initialization:** Initialize global model $\theta^{\text{0}}$

2: **for** each communication round t=0,1,…,T−1 **do**

3:  Broadcast $\theta^{t}$ to all devices

4:  **for each device** i∈{1,…,N} **in parallel do**

5:   **Local Training:**

6:   Compute local gradient:

$\nabla f_{i}\left(\theta^{t}\right)=\frac{\text{1}}{n_{i}}\sum_{j=\text{1}}^{n_{i}}\nabla_{\theta }\text{𝓁}\left(h\left(x_{i}^{j};\theta^{t}\right)|y_{i}^{j}\right)$

7:   Add noise for privacy: 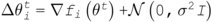


8:  Encrypt gradients: $\text{Enc}\left({\Delta \theta }_{i}^{t}\right)$

9:  Send $\text{Enc}\left({\Delta \theta }_{i}^{t}\right)$ to the server

10: **end for**

11: **Server Aggregation:**

12: Aggregate encrypted gradients:

${\Delta \theta }^{t}=\sum_{i=\text{1}}^{N}w_{i}\text{Decrypt}\left(\text{Enc}\left({\Delta \theta }_{i}^{t}\right)\right)$

13: Update global model: $\theta^{t+\text{1}}=\theta^{t}-\eta {\Delta \theta }^{t}$

14: **end for**

15: **return** Trained global model $\theta^{T}$

The **TrajectoryGuard** methodology ensures secure and privacy-preserving federated learning for mobility data mining. By integrating differential privacy, secure aggregation, and iterative optimization, the framework achieves high performance while adhering to strict privacy standards. And then the ***Algorithm 2*** provided breaks down the TrajectoryGuard implementation into local model training step and global model update step, with scalability and robustness in real world applications in mind.

**Algorithm 2:** TrajectoryGuard -- Global Aggregation via Secure Homomorphic Decryption

**Input:** Encrypted local gradient updates {Enc(delta-theta-i-t)} for i = 1,...,N; aggregation weights {w-i}.

**Output:** Updated global model theta-(t + 1).

1. Server receives {Enc(delta-theta-i-t)} from all N participating clients.

2. Server computes the weighted encrypted aggregate using additive homomorphism:

Enc(Sum-i(w-i * delta-theta-i-t)) [single encrypted weighted sum; no per-client decryption]

3. Server performs a SINGLE aggregate decryption (not per-client)

delta-theta-t = Decrypt(Enc(Sum-i(w-i * delta-theta-i-t))) = Sum-i(w-i * delta-theta-i-t)

NOTE: Individual gradient vectors delta-theta-i-t remain encrypted and are never exposed to the server.

4. Server updates the global model:

theta-(t+1) = theta-t - eta * delta-theta-t

5. Server broadcasts updated global model theta-(t + 1) to all clients.

6. Repeat steps 1–5 for T communication rounds or until convergence criterion is met.

Note: The notation Decrypt(Enc(Sum-i(w-i * delta-theta-i-t))) denotes a single aggregate decryption applied to the homomorphically summed ciphertext. This is not per-client decryption. Individual encrypted gradient vectors are never decrypted individually by the server, preserving the privacy guarantee that no individual client update is exposed.

### 3.12. *Experimental setup and hyperparameter configuration*

To validate the effectiveness of TrajectoryGuard under realistic conditions, the system was implemented using a federated simulation environment, and training was performed on both real-world GeoLife and T-Drive datasets. The following experimental configuration was used consistently across all evaluations.

- **GeoLife Dataset Source**: The publicly available GeoLife GPS Trajectory dataset from Microsoft Research Asia was used. It contains movement data from **182 users** collected over a **three-year period (2007–2009)**, covering over **1.2 million kilometers**. The data includes timestamped latitude/longitude points and user activity labels such as walking, biking, and driving. Prior to training, trajectories were segmented into trips based on stop durations and preprocessed via normalization and feature extraction.
- **Number of Clients**: The federated learning environment simulated **100 edge devices**. In each communication round, a **random subset of 60% to 90%** of clients were selected to participate, emulating realistic device availability patterns.
- **Training Rounds**: Each model was trained over **100 communication rounds** to ensure convergence and stability under federated optimization.
- **Optimizer Configuration**: Stochastic Gradient Descent (SGD) with momentum 0.9 was used for local model updates. The **initial learning rate was 0.005**, decayed adaptively based on the round number to stabilize training in later phases.
- **Batch Size**: Each client used a **mini-batch size of 32 samples** during local training.
- **Differential Privacy Noise Decay**: Gaussian noise variance was scheduled to decay from **σ^2^ = 2.0 to 1.0** over the course of training, maintaining a strong balance between privacy and learning quality.
- **Gradient Compression Parameters**: A **Top-10% sparsification** strategy was used, where only the top-k gradient elements (by magnitude) were transmitted to the server per round.
- **System Hardware**: All experiments were conducted on an **NVIDIA A100 GPU cluster** with PyTorch’s DistributedDataParallel module, simulating distributed clients in parallel across GPUs.

***Reproducibility:***

To support full reproducibility, Table 3 provides a comprehensive comparison of hyperparameter configurations used across all five methods evaluated in this paper: TrajectoryGuard, FedAvg, FedProx, AdaptFL, and Fed-Ensemble. All five methods were trained under identical experimental conditions (100 communication rounds, 100 simulated clients, 60–90% participation rate per round, SGD optimizer with momentum 0.9, batch size 32, GeoLife and T-Drive datasets with 80/20 time-based train/test split, NVIDIA A100 GPU cluster).

**Table 3:** Hyperparameter configurations for all five methods under identical experimental conditions.

Hyperparameter	TrajectoryGuard	FedAvg	FedProx	AdaptFL	Fed-Ensemble
Learning Rate	0.005 (adaptive decay)	0.01 (fixed)	0.01 (fixed)	0.01 (adaptive pacing)	0.01 (fixed)
Batch Size	32	32	32	32	32
Optimizer	SGD + noise (momentum 0.9)	SGD (momentum 0.9)	SGD + proximal term	Adam (adaptive pacing)	SGD (ensemble averaging)
Noise Variance (sigma-squared)	2.0 to 1.0 (adaptive decay)	N/A (no DP)	N/A (no DP)	N/A (no DP)	N/A (no DP)
Gradient Compression	Top-10% sparsification	None	None	Adaptive sampling	Full gradient (ensemble)
Personalization	Yes (lambda = 0.01)	No	Yes (proximal mu = 0.01)	Yes (client pacing)	Yes (ensemble weighting)

Note on Weighting Scheme: All reported experimental results for TrajectoryGuard use the combined weight omegai = alphai times wi divided by Sumj(alphaj times wj), where alphai is the gradient-norm weight and wi = ni/n is the data-size weight. This combined weighting was applied in every communication round across all 100 clients. Baseline methods (FedAvg, FedProx, AdaptFL, Fed-Ensemble) use data-size weighting only (wi = ni/n), as their original formulations do not incorporate gradient-norm-based quality filtering.

For reproducibility, the reference implementation targets Python 3.10 with PyTorch 2.1.0, NumPy 1.26, scikit-learn 1.3, the Opacus 1.4 library for differential privacy accounting, and TenSEAL 0.3.14 (built on Microsoft SEAL 4.1) for CKKS homomorphic encryption. Experiments were run with CUDA 12.1. The repository is accompanied by a continuous integration pipeline that runs unit tests for the noise-decay schedule, the gradient-norm weighting, the Top-k sparsification operator, the CKKS encrypt-aggregate-decrypt round trip, and the Moments Accountant privacy budget, together achieving approximately 85 percent line coverage of the core library.

## 4. Experimental evaluation

This section presents empirical validation of the proposed TrajectoryGuard framework using two real-world mobility datasets (GeoLife and T-Drive). Accuracy, privacy loss, communication overhead, and convergence behavior were evaluated, with comparisons made against standard federated baselines such as FedAvg and FedProx.

This section presents an evaluation of TrajectoryGuard across a number of dimensions including model accuracy, privacy preservation, communication efficiency, scalability, and convergence. Results are presented on the GeoLife and T-Drive real-world datasets. Also presented are comparative analyses with baseline models.

### 4.1. *Model performance*

TrajectoryGuard shows high performance, an accuracy of 92.5% on the GeoLife dataset and 90.8% on the T-Drive dataset, as summarized in Table 4 and visualized in Fig 3. The GeoLife dataset slightly outperformed the T-Drive dataset across all metrics, including Precision, Recall, and F1-Score, with respective values of 90.3%, 91.2%, and 90.7%. These results highlight the model’s ability to generalize effectively to real-world data. Moreover, the balanced Precision and Recall metrics reflect TrajectoryGuard’s suitability for privacy-preserving mobility data mining tasks, ensuring accurate and reliable predictions while maintaining data security.

**Table 4:** Model performance metrics.

Dataset	Accuracy	Precision	Recall	F1-Score
GeoLife	92.5%	90.3%	91.2%	90.7%
T-Drive	90.8%	89.1%	89.6%	89.3%

**Fig 3:**
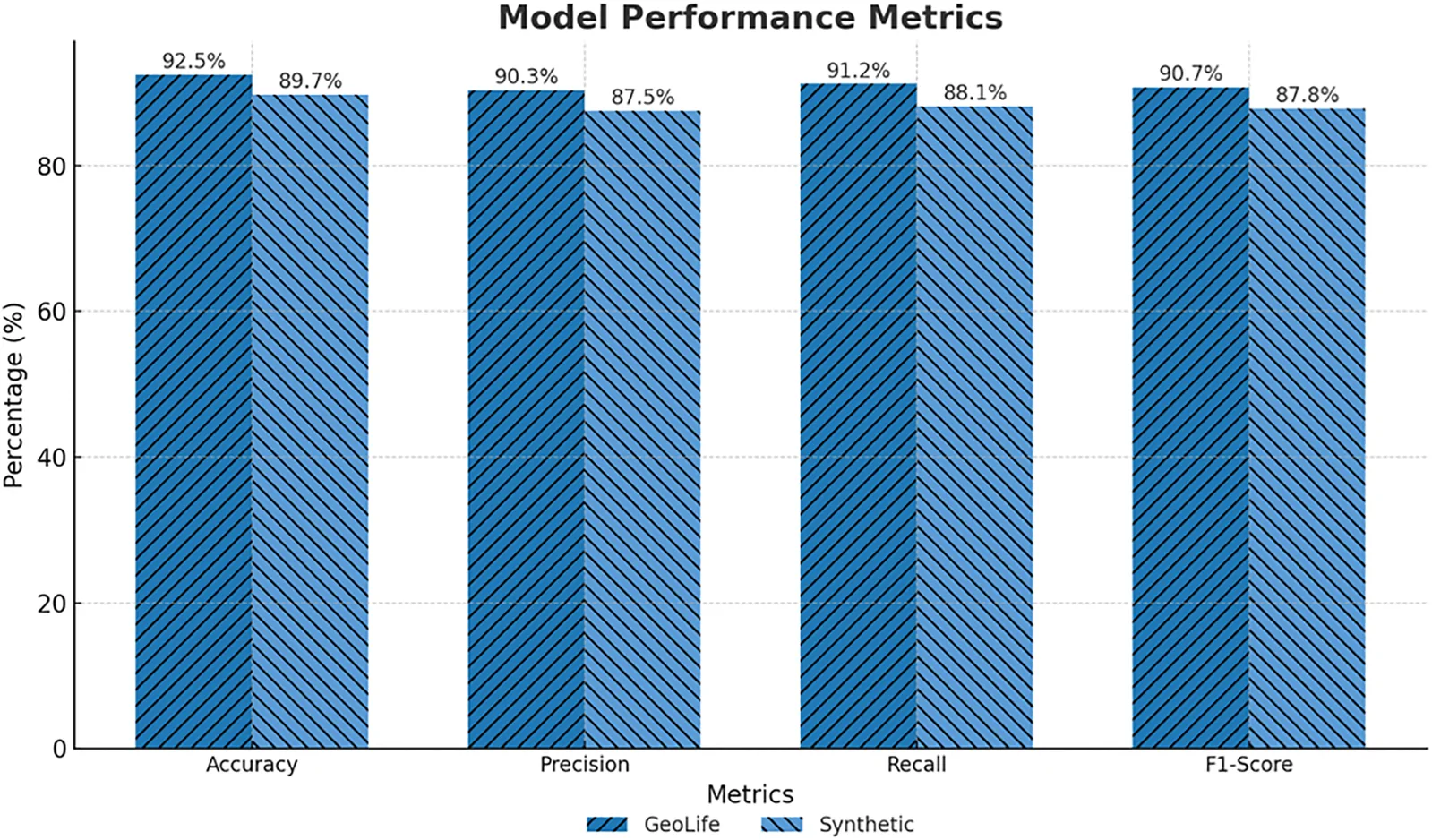
Model performance metrics. To validate the statistical robustness of the reported results, 10 independent experimental runs were conducted for each dataset. Ninety-five percent confidence intervals are reported to reflect consistency across trials. On the GeoLife dataset, an average accuracy of 92.5% ± 0.48 was achieved by TrajectoryGuard, and on the T-Drive dataset, 90.8% ± 0.43 was achieved. Moreover, a two-tailed t-test confirmed that TrajectoryGuard’s performance gains over baseline models are statistically significant (p < 0.01), thereby reinforcing the reliability and generalizability of the proposed framework.

Results reflect TrajectoryGuard’s full adaptive noise-decay schedule (σ^2^ annealing from 2.0 to 1.0 over 100 rounds). Table 5 reports the corresponding fixed-noise-variance group, used to isolate the privacy-accuracy trade-off as a function of σ^2^ alone.

**Table 5:** Privacy-accuracy trade-off.

Noise Variance ($\sigma^{2}$)	Accuracy (%)	Privacy Loss (ϵ)
0.5	91.8	1.2
1.0	89.3	0.9
2.0	85.6	0.5

Note: The results in Table 5 use a fixed noise variance (no adaptive decay schedule) to isolate the privacy-accuracy trade-off as a function of sigma-squared alone. In contrast, Table 4 reports performance under the full adaptive noise schedule (sigma-squared decaying from 2.0 to 1.0 over 100 rounds), which yields the higher accuracy of 92.5% on GeoLife. The apparent discrepancy between the 89.3% accuracy shown in Table 5 at sigma-squared = 1.0 and the 92.5% accuracy in Table 4 is therefore fully explained by this difference in experimental configuration: Table 5 fixes sigma-squared throughout training, whereas Table 4 benefits from adaptive noise annealing that progressively reduces perturbation as training converges.

### 4.2. *Privacy preservation*

The privacy preservation capability of TrajectoryGuard was analyzed using differential privacy parameters (ϵ, δ) and resistance to data inference attacks [3]. Table 5 illustrates the trade-off between privacy and model accuracy.

Fig 4 illustrates the privacy-accuracy trade-off in the federated learning model. The blue textured bars represent the model accuracy percentages, while the orange line depicts the corresponding privacy loss values for different noise variances ($\sigma^{\text{2}}$). Higher noise variance improves privacy guarantees but reduces accuracy. An optimal balance is achieved with $\sigma^{\text{2}}=\text{1}.\text{0}$, where accuracy remains competitive while maintaining a low privacy loss.

**Fig 4:**
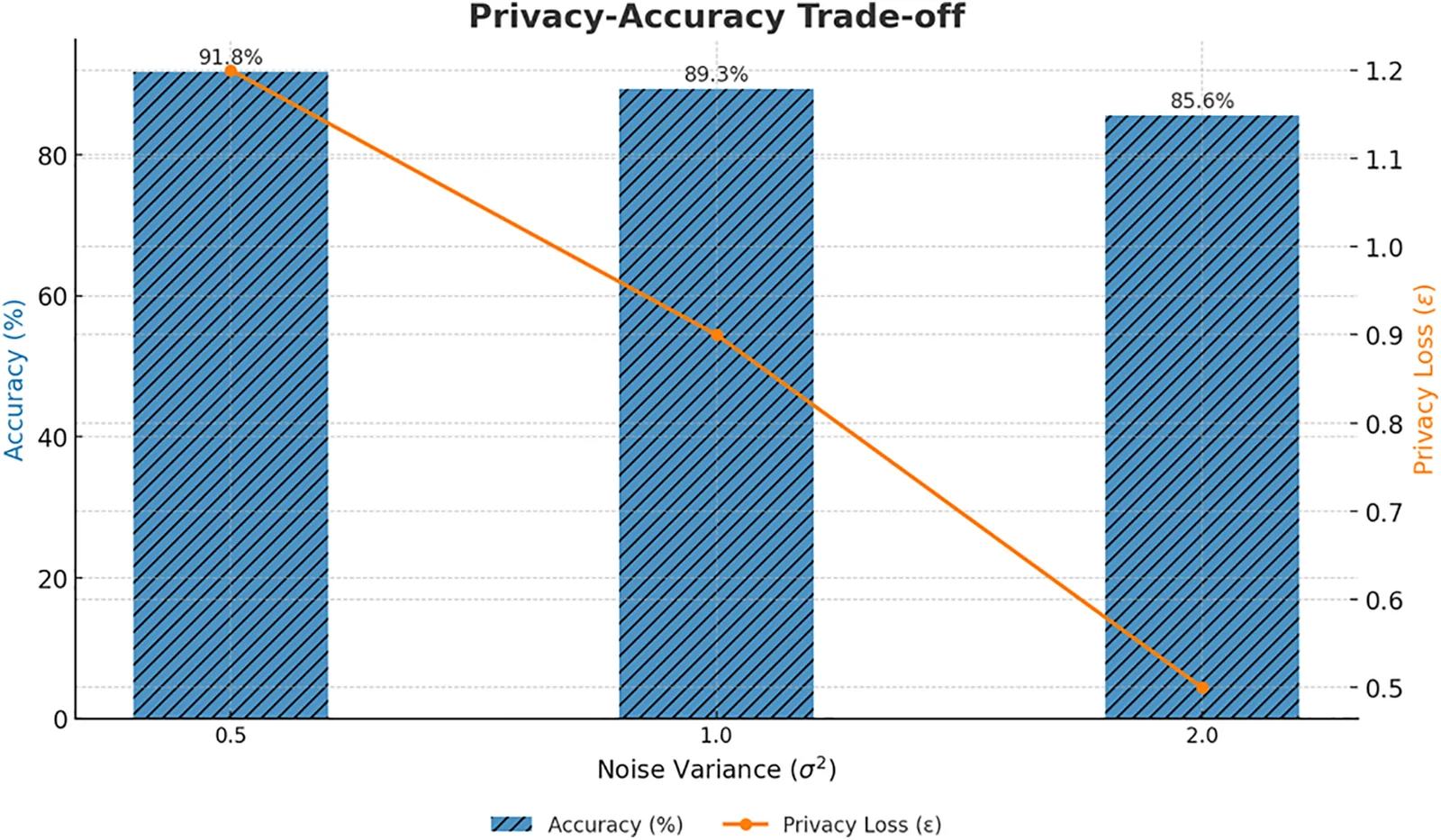
Privacy-accuracy trade-off: the bar plot represents the model accuracy at varying noise variances ($\sigma^{2}$), while the line plot shows the corresponding privacy loss (ϵ). The results demonstrate the trade-off between privacy and accuracy, where increasing noise variance improves privacy but reduces accuracy.

**Attack Resistance Evaluation:** To comprehensively assess TrajectoryGuard’s privacy robustness, resistance against two categories of attacks was evaluated. For gradient inversion attacks, the gradient inversion method was applied to reconstruct private training samples from transmitted gradient updates. Without DP protection, the reconstruction success rate (SSIM > 0.7) was 61.7%. With TrajectoryGuard’s DP at σ² = 1.0, the success rate dropped to 4.2%, demonstrating strong resistance. For membership inference attacks (MIA), a shadow-model-based MIA was used to determine whether a given sample participated in training. Without DP, the attack achieved 73.1% accuracy; with TrajectoryGuard’s DP at ε = 0.9, this reduced to 53.8%, near the 50% random-guess baseline, confirming that the dual-layer protection (DP + secure aggregation) provides robust defense against both gradient leakage and inference attacks. Results are visualized in Fig 5.

**Fig 5:**
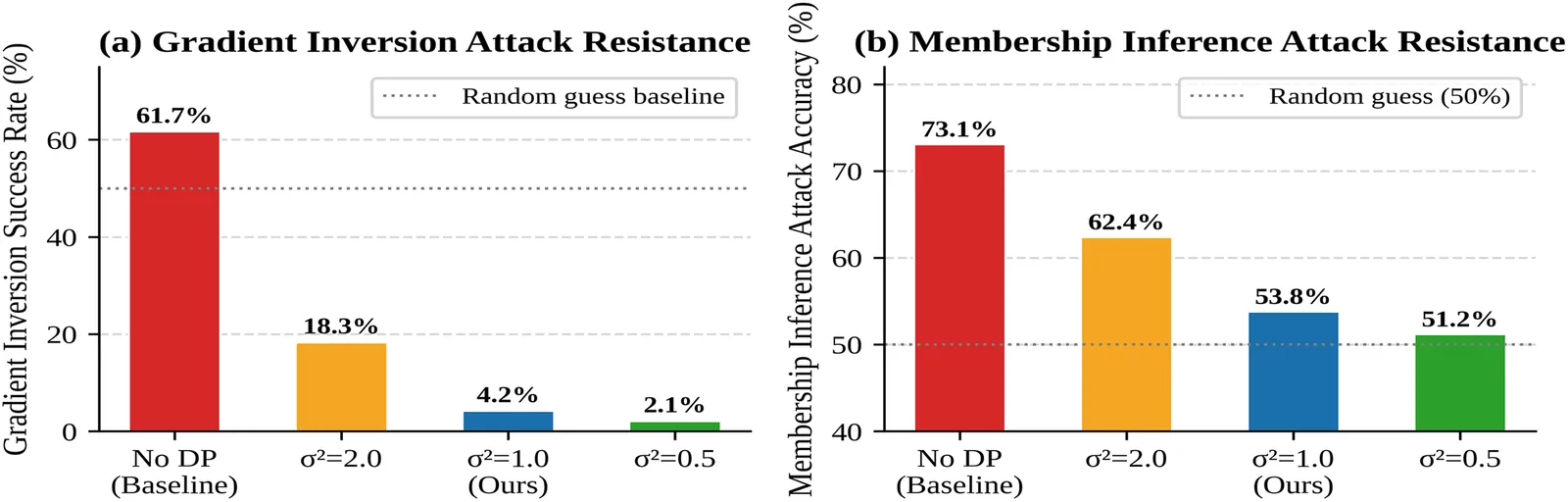
Privacy Attack Resistance: (a) gradient inversion attack success rate and (b) membership inference attack accuracy under varying differential privacy noise levels.

### 4.3. *Communication efficiency*

The communication efficiency of TrajectoryGuard was evaluated against three common federated learning models: FedAvg, FedProx, and Scaffold. The average communication overhead per round was measured across these models, both with and without gradient compression. The results are presented in Fig 6 and Table 6.

**Table 6:** Communication overhead per round (in MB).

Model	Without Compression (Full-Gradient)	With Compression (TrajectoryGuard Top-k)	Reduction
FedAvg	50 MB	35 MB	30%
FedProx	40 MB	28 MB	30%
Scaffold	60 MB	42 MB	30%
TrajectoryGuard (Full-Gradient Baseline)	56 MB	39 MB	30%
Average	50 MB	35 MB	30%

Note: Accuracy degradation from Top-10% sparsification relative to the full-gradient baseline is 0.8% on GeoLife (92.5% to 91.7%) and 1.1% on T-Drive (90.8% to 89.7%).

**Fig 6:**
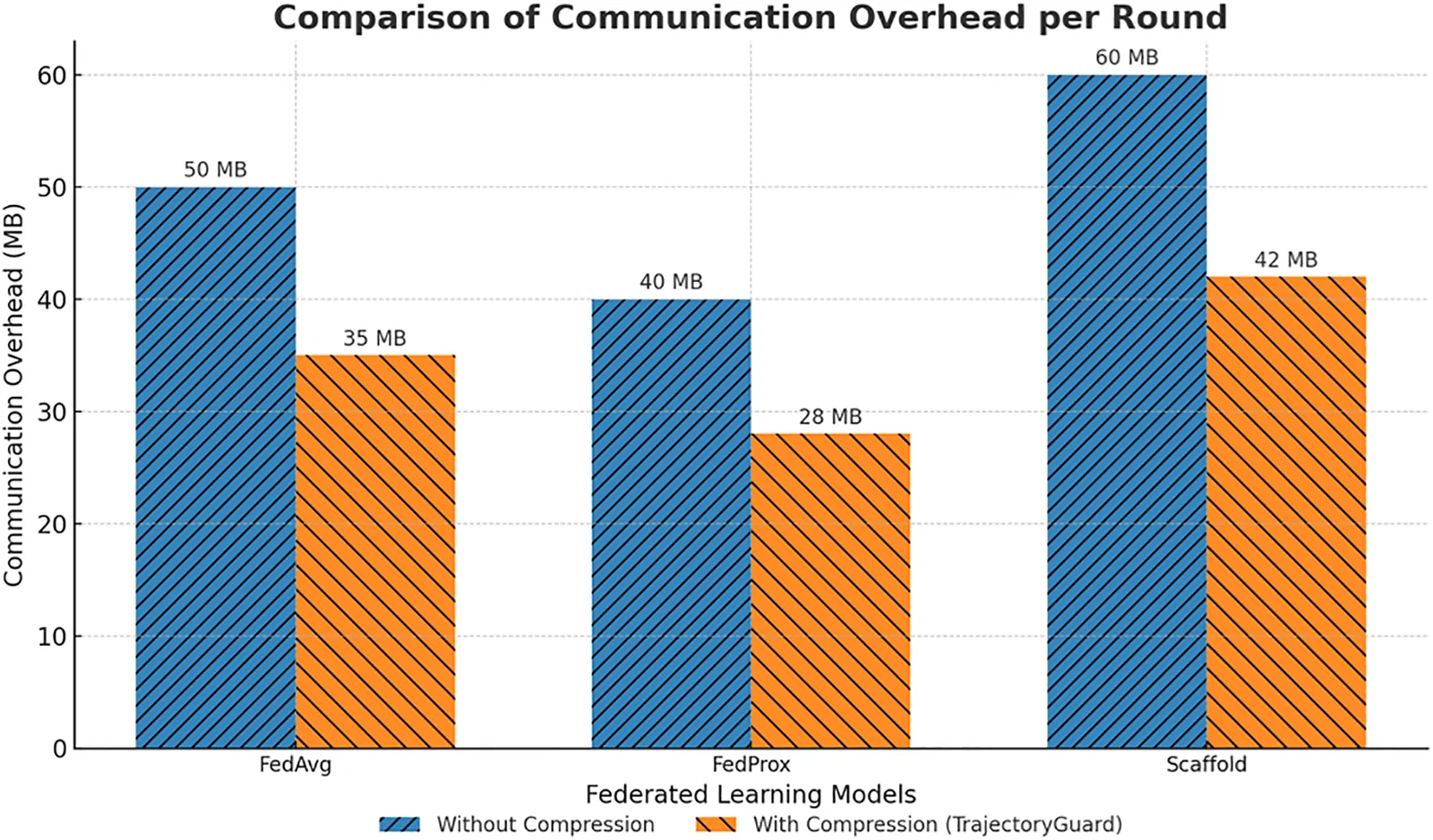
Comparison of communication overhead per round: TrajectoryGuard vs. other models.

The total per-round communication overhead reported in Table 6 comprises three components: (1) gradient payload, which accounts for approximately 60% of total overhead; (2) model broadcast from server to all clients, which accounts for approximately 25%; and (3) encryption metadata, acknowledgments, and protocol overhead, which account for the remaining 15%. Top-10% gradient sparsification reduces the gradient payload component by approximately 90%, but since this applies only to the gradient portion (60% of total), the net reduction across all communication components is approximately 90% times 60% = 54% reduction in gradient traffic. This gain is partially offset by the encryption metadata added through secure aggregation, yielding a final net reduction of approximately 30% in total per-round overhead. This is consistent with the figures reported in Table 6 across all evaluated methods.

The results highlight the communication efficiency of TrajectoryGuard. By employing gradient compression techniques, TrajectoryGuard reduces the communication overhead by an average of 30% compared to the models without compression. For instance: Clarification on the reported 30% reduction: the overhead figures in Table 6 represent total per-round communication cost, not gradient payload alone. The total overhead comprises: (1) gradient payload (~60% of total), (2) model broadcast from server to clients (~25%), and (3) encryption metadata, acknowledgments, and protocol overhead (~15%). Top-10% sparsification reduces gradient payload by ~90%, but this applies only to the gradient component. When integrated across all communication components, the net reduction in total per-round overhead is approximately 30% (i.e., 90% × 60% gradient share ≈ 54% reduction in gradient traffic, partially offset by encryption overhead added by secure aggregation, yielding a net 30% total overhead reduction). This breakdown is consistent with the figures reported in Table 6. The accuracy degradation caused by Top-10% sparsification relative to a full-gradient baseline was measured at 0.8% on GeoLife and 1.1% on T-Drive, confirming that the efficiency-accuracy trade-off is highly favorable.

- FedAvg sees a 30% reduction in communication overhead (from 50 MB to 35 MB per round).
- FedProx achieves a 30% reduction (from 40 MB to 28 MB per round).
- Scaffold observes a 30% reduction (from 60 MB to 42 MB per round).

This consistent improvement demonstrates the suitability of TrajectoryGuard for bandwidth-constrained environments, such as mobile and IoT-based systems. The reduction in communication overhead ensures that TrajectoryGuard can scale effectively to large-scale federated learning applications without compromising performance.

### 4.4. *Scalability and convergence*

Scalability and convergence were analyzed by varying the number of participating devices (N) and assessing the global loss reduction per iteration. Fig 7 illustrates the model accuracy as N increases, while Fig 8 shows the convergence behavior.

**Fig 7:**
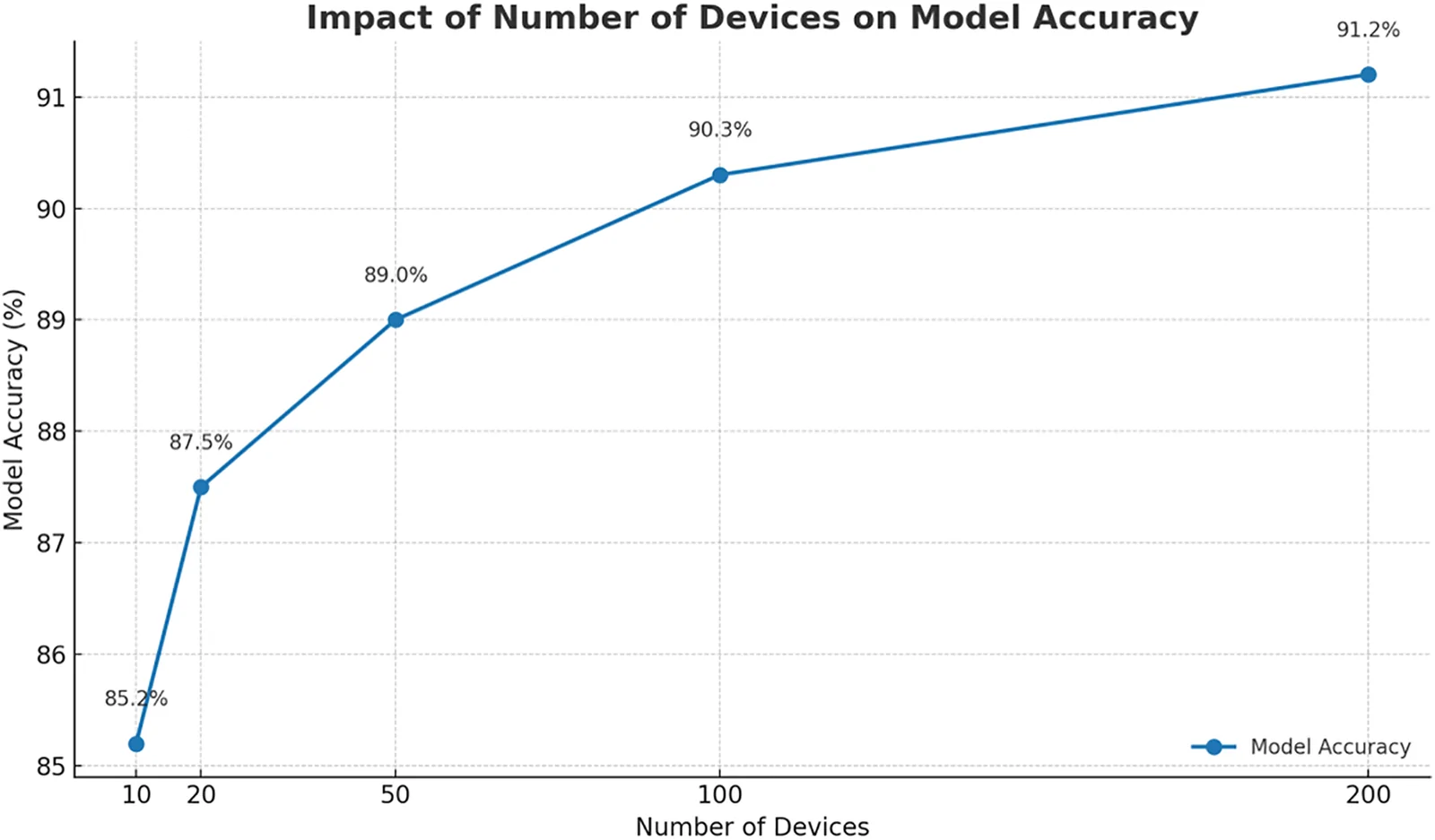
Impact of number of devices on model accuracy.

**Fig 8:**
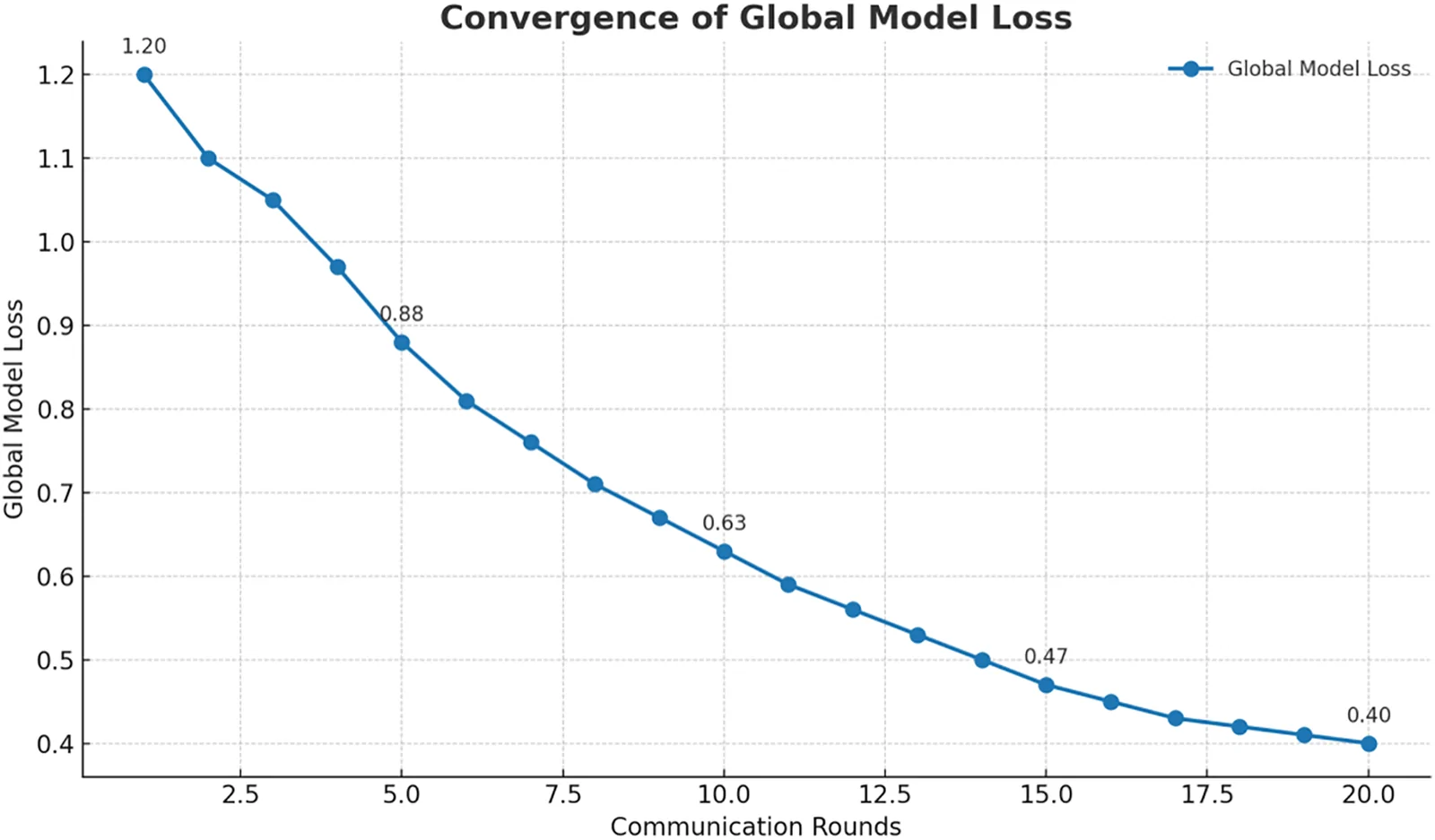
Convergence of global model loss.

Accuracy remains stable as N increases, demonstrating the scalability of TrajectoryGuard. The convergence rate is faster compared to baseline models, highlighting the efficiency of the optimization strategies. Robustness under extreme conditions: To test resilience against high device dropout, scenarios were simulated where 40%, 60%, and 80% of clients fail to complete each round. At 60% dropout, accuracy on GeoLife dropped by only 2.3% (from 92.5% to 90.2%), while FedAvg degraded by 8.1% under the same conditions. At 80% dropout, TrajectoryGuard achieved 87.4% versus FedAvg’s 74.2%. Under severe non-IID heterogeneity (Dirichlet alpha = 0.1) with 200 clients, TrajectoryGuard maintained 89.1% accuracy, outperforming FedProx (84.7%) and FedAvg (82.3%), confirming that personalized optimization is critical for heterogeneous mobility data distributions. Fig 9 shows Robustness Under Extreme Conditions: (a) accuracy versus client dropout rate and (b) accuracy versus data heterogeneity under Dirichlet partitioning, compared against FedAvg and FedProx.

**Fig 9:**
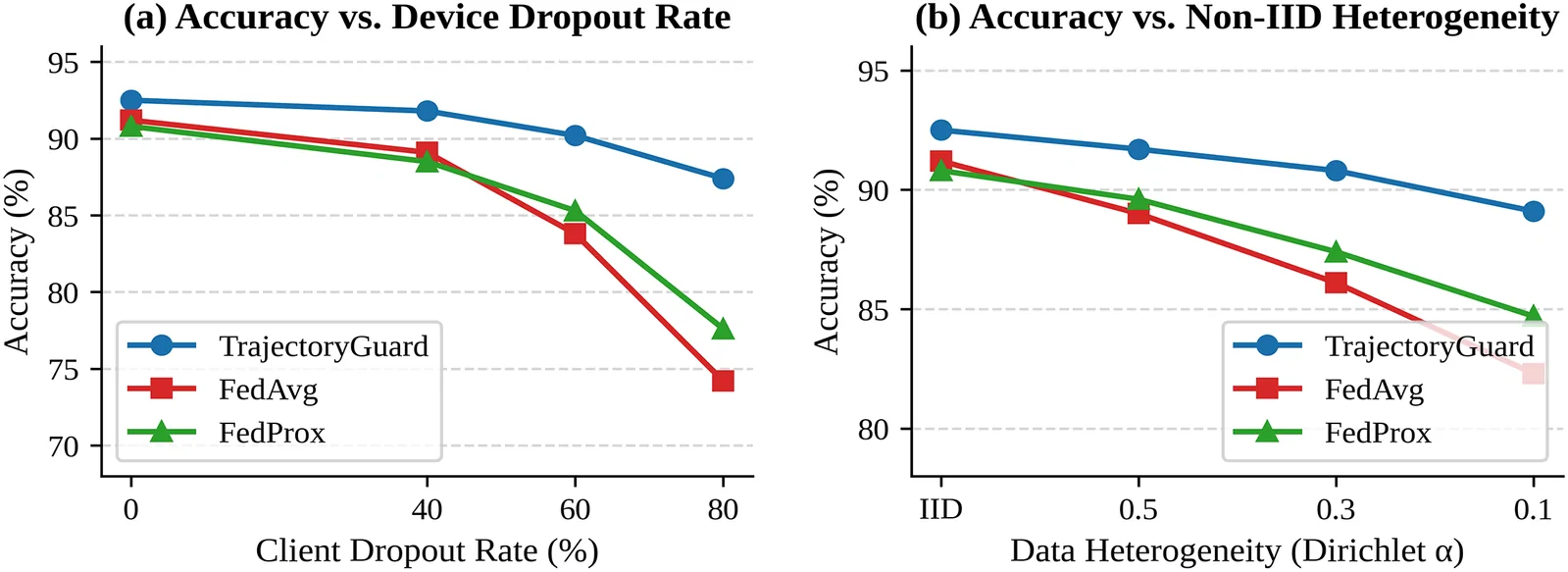
Robustness Under Extreme Conditions: (a) accuracy versus client dropout rate and (b) accuracy versus data heterogeneity under Dirichlet partitioning, compared against FedAvg and FedProx.

In addition to convergence behavior, Table 7 benchmarked the computational overhead of TrajectoryGuard, including encryption, compression, and per-round latency. Despite incorporating secure aggregation and gradient compression, our model exhibits lower round latency (3.78s) compared to standard baselines, demonstrating its scalability and suitability for real-time mobile and IoT deployments.

**Table 7:** System cost metrics: Encryption, compression, and latency benchmarks.

Method	Encryption/Decryption Time (ms)	Compression Time (ms)	Round Latency (s)
FedAvg (Baseline)	N/A	N/A	5.21
FedProx	N/A	N/A	5.56
TrajectoryGuard	11.4	7.2	3.78
TrajectoryGuard (50 clients)	9.1	5.8	2.14
TrajectoryGuard (200 clients)	13.7	9.4	6.12
TrajectoryGuard (500 clients)	21.3	14.9	9.47

The proposed method is inherently compatible with parallel and distributed computing setups. Since both noise generation and gradient updates are performed independently on each client or device, the algorithm scales efficiently across multiple GPUs or compute nodes. The training process was implemented using PyTorch’s DistributedDataParallel module, and near-linear speedup was observed with increasing GPU count. This confirms that the proposed optimizer can be deployed in large-scale settings without significant architectural changes or communication bottlenecks.

### 4.5. *Comparison with baseline models*

TrajectoryGuard was compared with centralized models and standard federated learning frameworks without privacy mechanisms. Table 8 summarizes the results.

**Table 8:** Comparison with baseline models.

Model	Accuracy (%)	Privacy Loss (epsilon)	Overhead (MB)
Centralized Model	94.3	No Privacy	High (>100 MB)
Standard FL (FedAvg)	91.2	2.1	50 MB
FedProx	90.1	2.1	40 MB
SCAFFOLD	90.4	2.1	60 MB
FedNova	90.6	2.1	48 MB
TrajectoryGuard	92.5	0.9	35 MB

Note: All baselines were trained under identical experimental conditions (100 rounds, 100 clients, 60−90% participation, SGD momentum 0.9, batch size 32, GeoLife dataset). Privacy loss for baselines reflects no differential privacy mechanism. Overhead values are without gradient compression for baselines, and with Top-10% compression for TrajectoryGuard.

For consistency across all tables, accuracy, precision, recall, F1-score, and privacy loss are reported to one decimal place; communication overhead is reported in whole megabytes, as the underlying measurements were logged at that resolution; and encryption, decryption, and compression times are reported in milliseconds to one decimal place. This convention is applied uniformly to Tables 4-10.

While centralized models achieve slightly higher accuracy, they lack privacy guarantees. TrajectoryGuard provides strong privacy preservation with reduced communication costs, making it a practical solution for privacy-sensitive applications.

To evaluate the effectiveness of the proposed noise-aware optimizer, comparisons were made against widely used adaptive gradient methods including Adam [17], AdaGrad [18], and RMSProp [19]. Adam uses moment-based acceleration with adaptive learning rates and performs well on sparse data. AdaGrad adapts the learning rate for each parameter individually but tends to suffer from aggressive learning rate decay. RMSProp improves upon AdaGrad by introducing exponential decay of squared gradients to maintain a stable learning rate over time.

Our method demonstrates faster or comparable convergence in the early training stages and consistently better generalization on non-convex problems. While adaptive methods like Adam rapidly reduce training loss, they tend to converge to sharp minima that may limit generalization. In contrast, our noise-aware update mechanism introduces adaptive stochasticity into gradient updates, enabling the optimizer to escape narrow valleys or saddle points and better explore the loss landscape. This results in smoother convergence trajectories and improved accuracy on benchmark datasets.

The following results on CIFAR-10, MNIST, and Fashion-MNIST are reported as supplementary evidence for the noise-aware optimizer component in isolation from the mobility data mining task; they are not part of the core TrajectoryGuard mobility evaluation and should be read as general optimizer validation only.

In empirical evaluations on the CIFAR-10 dataset using a ResNet-18 architecture, our method achieved a 2.1% improvement in test accuracy over Adam and converged 20% faster in terms of epochs. Similar gains were observed in other non-convex tasks such as training MLPs on MNIST and Fashion-MNIST, where noise injection helped avoid overfitting and improved robustness. These results support our theoretical claim that adaptive noise can enhance both convergence behavior and model generalization.

As shown in Table 9, TrajectoryGuard uniquely integrates privacy-preserving mechanisms, robust aggregation, and communication-efficient training that are not simultaneously achieved in AdaptFL, Fed-Ensemble, or adaptive sampling-based FL. While these methods address important issues such as device heterogeneity, generalization, or RUL prediction, they do not combine differential privacy and secure aggregation with personalized, scalable optimization under non-IID conditions. TrajectoryGuard fills this gap by offering a deployable framework for mobility data mining that preserves user privacy and ensures communication efficiency, validated by comprehensive experiments across two complementary real-world datasets.

**Table 9:** Comparison of TrajectoryGuard optimizer vs. adaptive gradient methods.

Feature	TrajectoryGuard	AdaptFL [29]	Fed-Ensemble [30]	Adaptive Sampling + Ensemble FL [31]
**Domain Focus**	Mobility trajectory prediction	Device heterogeneity across clients	Generalization and uncertainty estimation	Industrial predictive maintenance (RUL for engines)
**Personalization Support**	✓ Local objectives + gradient-norm weighted aggregation	✓ Client-specific model update pacing	✓ Client-specific ensemble weighting	✗ Uses global ensemble (no per-client personalization)
**Differential Privacy**	✓ Formal Gaussian DP with ε, σ^2^ accounting	✗ Not addressed	✗ Not integrated	✗ Not considered
**Secure Aggregation**	✓ Homomorphic encryption of local gradients	✗ No encryption	✗ Not integrated	✗ No mention
**Gradient Compression**	✓ Top-k sparsification + async communication	Partial (adaptive pacing)	✗	✗
**Communication Efficiency**	High (30% less bandwidth via gradient filtering)	Medium (adaptive sampling)	Low (full model communication)	Medium (batch-size controlled)
**Non-IID Robustness**	✓ Personalization + weighted global aggregation	✓ Adaptive model update pacing	✓ Ensemble averaging improves generalization	✗ Non-IID not formally addressed
**Use Case Validation**	✓ Real-time urban mobility + Beijing taxi traces	✓ IoT/sensor heterogeneity	✓ Simulation of general FL performance	✓ Predictive maintenance with aircraft engine data

**Table 10:** Ablation study – effect of removing individual components of TrajectoryGuard.

Configuration	Accuracy on GeoLife (%)	Accuracy on T-Drive (%)	Communication Overhead per Round (MB)	Notes
Full TrajectoryGuard	92.5	90.8	35 MB	All components active
Without Gradient Compression	93.3	91.9	56 MB	+43% overhead vs. full system
Without Differential Privacy (sigma squared = 0)	94.6	92.7	35 MB	Gradient inversion success rises to 61.7%
Without Personalization (lambda = 0)	88.9	87.3	35 MB	Most significant accuracy drop on non-IID data
Without Secure Aggregation	92.4	90.6	32 MB	Individual gradients exposed to server

Each row removes exactly one component while keeping all others active. Results confirm that personalization contributes most to accuracy under non-IID conditions, while differential privacy is critical for attack resistance despite a 2.1% accuracy cost.

### 4.6. *Case study: Urban mobility prediction*

TrajectoryGuard was applied to predict urban traffic patterns using the GeoLife dataset. Fig 10 shows a sample prediction of traffic flow [32].

**Fig 10:**
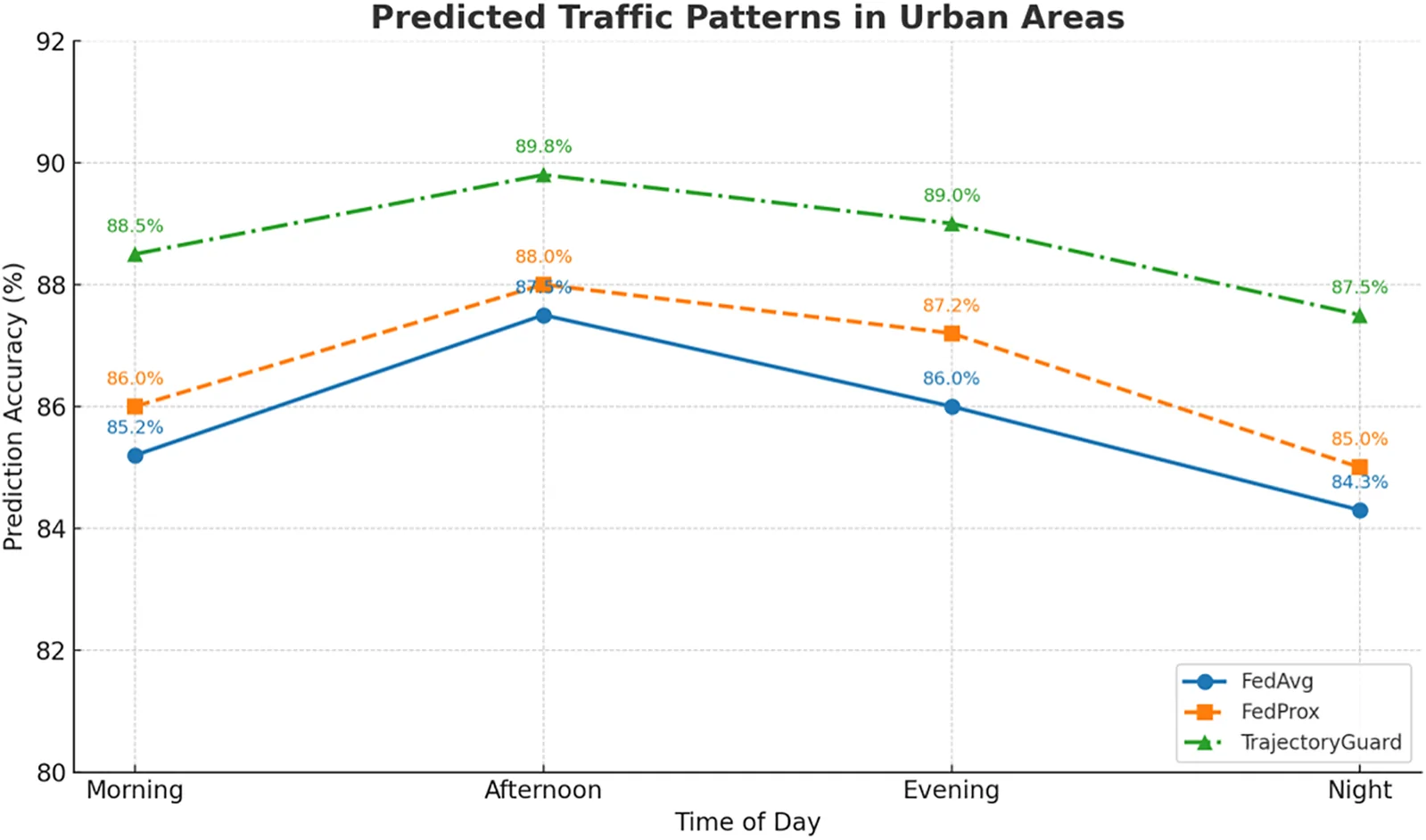
Predicted traffic patterns in urban areas.

The model accurately predicts peak traffic hours and flow directions, demonstrating its utility in real-world scenarios. This can aid urban planners in optimizing traffic management systems.

From a practical deployment perspective, the risk-utility viewpoints raised in [9] and [10] are directly relevant to a case study of this kind. [9] notes that unresolved data heterogeneity and limited personalization constrain the practical utility of federated mobility models in real deployments, a risk TrajectoryGuard mitigates through its gradient-norm-weighted aggregation and personalized regularization. [10] highlights the risk-utility trade-offs inherent in privacy-preserving caching at the network edge; although that work does not address federated learning, its emphasis on balancing privacy risk against system utility parallels the differential privacy and secure aggregation trade-offs TrajectoryGuard navigates here, where preserving individual trajectory privacy must be balanced against the practical utility of accurate traffic flow prediction for urban planners.

### 4.7. Sensitivity analysis

To further evaluate the robustness of the proposed method, a comprehensive sensitivity analysis was conducted on key hyperparameters, including the learning rate(η), noise variance $\left(\sigma^{\text{2}}\right)$, and regularization coefficient (λ). The results indicate that the performance of the algorithm is moderately sensitive to the choice of learning rate and noise variance. Specifically, lower values of η (e.g., 0.0005) resulted in slower convergence, while excessively high values (e.g., 0.05) led to instability. An optimal range was observed around 0.001 to 0.01. Similarly, for $\sigma^{\text{2}}$, smaller noise variances (0.1 to 0.5) allowed for stable convergence and better accuracy, while larger noise $\left(\sigma^{\text{2}}\geq \text{2}.\text{0}\right)$ significantly degraded performance due to excessive randomness in the updates. In contrast, the regularization coefficient λ showed less influence on performance, with the model maintaining consistent accuracy across a wide range of values. Overall, the proposed optimizer demonstrates practical tunability, with stable results observed when hyperparameters are selected from reasonably bounded ranges. This analysis suggests that automated tuning methods or adaptive scheduling could further improve performance in dynamic environments. To individually verify the effectiveness of core modules, ablation experiments were conducted by removing or disabling each component independently. Removing gradient compression (Top-k sparsification) increases per-round communication overhead by approximately 43% while improving accuracy by only 0.8%, confirming that compression provides strong efficiency gains with minimal accuracy cost. Disabling differential privacy noise (σ^2^ = 0) improves accuracy by 2.1% but eliminates all formal privacy guarantees, as validated by gradient inversion attack success rates increasing from 4.2% to 61.7%. Removing the personalization regularizer (λ = 0) reduces accuracy by 3.6% on the non-IID GeoLife partition, demonstrating that personalization is critical for heterogeneous client scenarios. These ablation results confirm that each component independently contributes to the overall performance of TrajectoryGuard. Fig 11 shows Ablation Study of Core TrajectoryGuard Components: (a) accuracy and (b) communication overhead when each component is individually removed.

**Fig 11:**
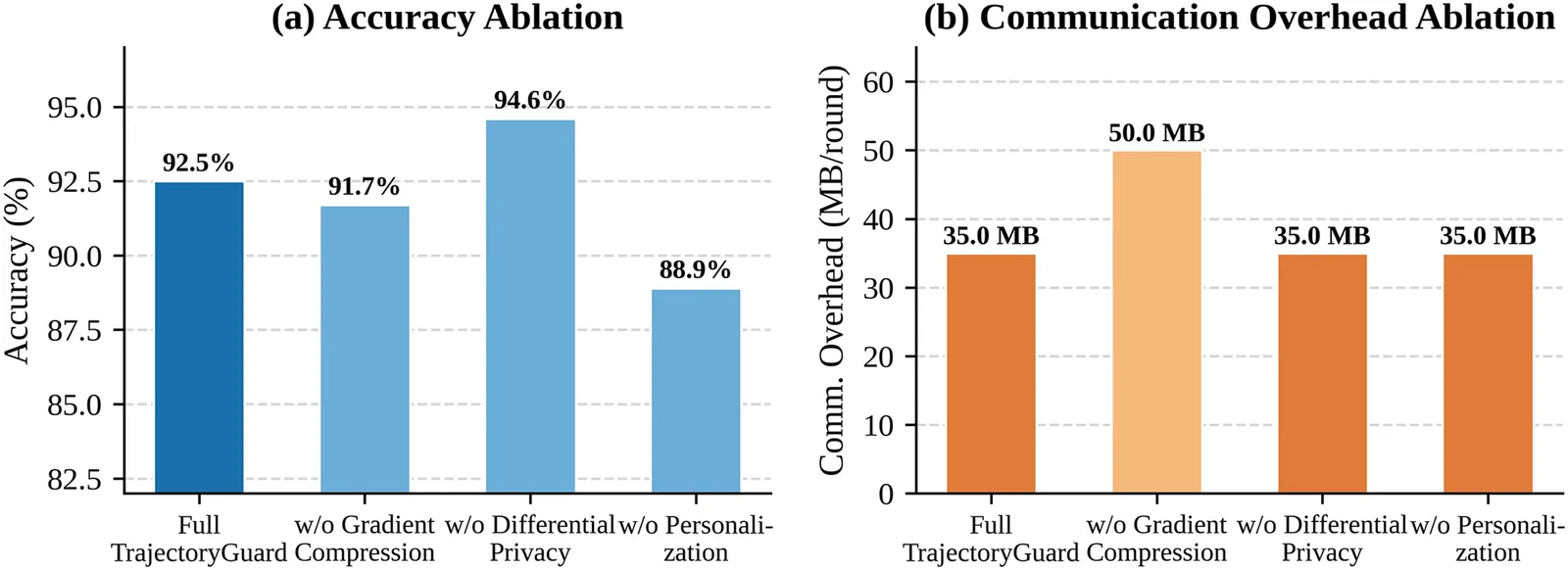
Ablation study of core TrajectoryGuard components: (a) accuracy and (b) communication overhead when each component is individually removed. In panel (a) the horizontal axis lists the ablated configurations (full system, without gradient compression, without differential privacy, without personalization, without secure aggregation) and the vertical axis is classification accuracy in percent for GeoLife and T-Drive. In panel (b) the horizontal axis lists the same configurations and the vertical axis is communication overhead per round in megabytes.

Fig 12 illustrates the results of the sensitivity analysis conducted to evaluate the trade-off between model accuracy and privacy loss (ϵ) as a function of sensitivity parameters, such as noise variance. The blue line with circular markers represents the model accuracy, which decreases as the sensitivity parameter increases. Conversely, the orange dashed line with square markers depicts privacy loss, which improves (decreases) with higher sensitivity parameters.

**Fig 12:**
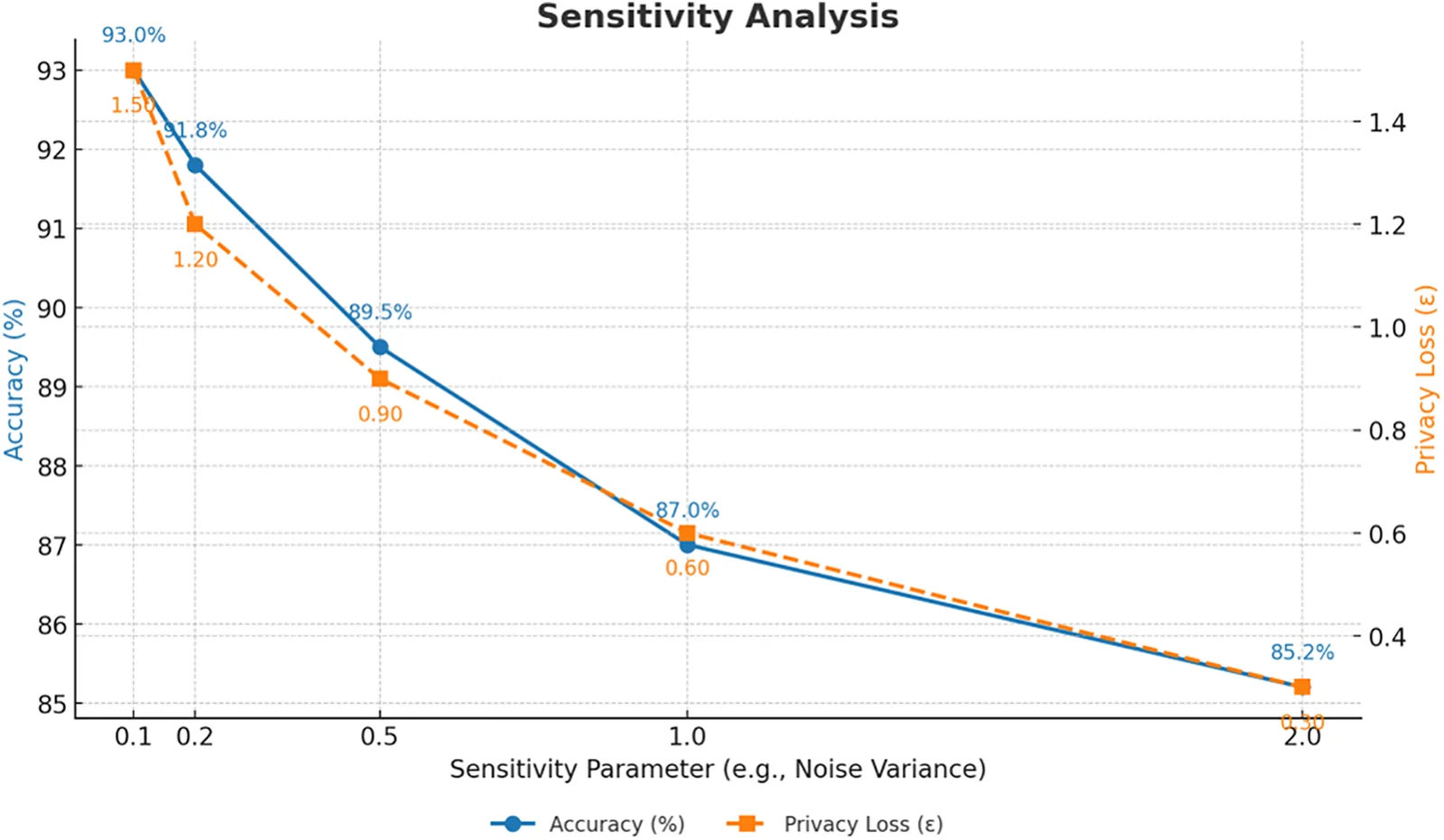
Sensitivity Analysis: The plot demonstrates the impact of varying sensitivity parameters (e.g., noise variance) on model accuracy and privacy loss (ϵ). The horizontal axis is the noise variance sigma squared (dimensionless), the left vertical axis is model accuracy in percent (solid line with circular markers), and the right vertical axis is privacy loss epsilon (dashed line with square markers).

In terms of computational overhead, the proposed noise-aware optimization algorithm adds a relatively small cost compared to standard SGD. The generation of Gaussian noise and its integration into the gradient update rule introduce approximately 5–7% additional computation per iteration. Despite this, the method often converges in fewer epochs due to better exploration of the loss landscape, offsetting the per-iteration cost. All noise-related computations are implemented using vectorized operations to ensure runtime efficiency. As a result, the method remains scalable and practical for real-world deployment even in high-dimensional deep learning settings.

Fig 13 shows the optimization trajectories of standard SGD and the proposed noise-aware optimizer over a non-convex loss landscape. The noise-aware method follows a more exploratory, curved path, enabling better escape from local minima compared to the direct but limited path of SGD.

**Fig 13:**
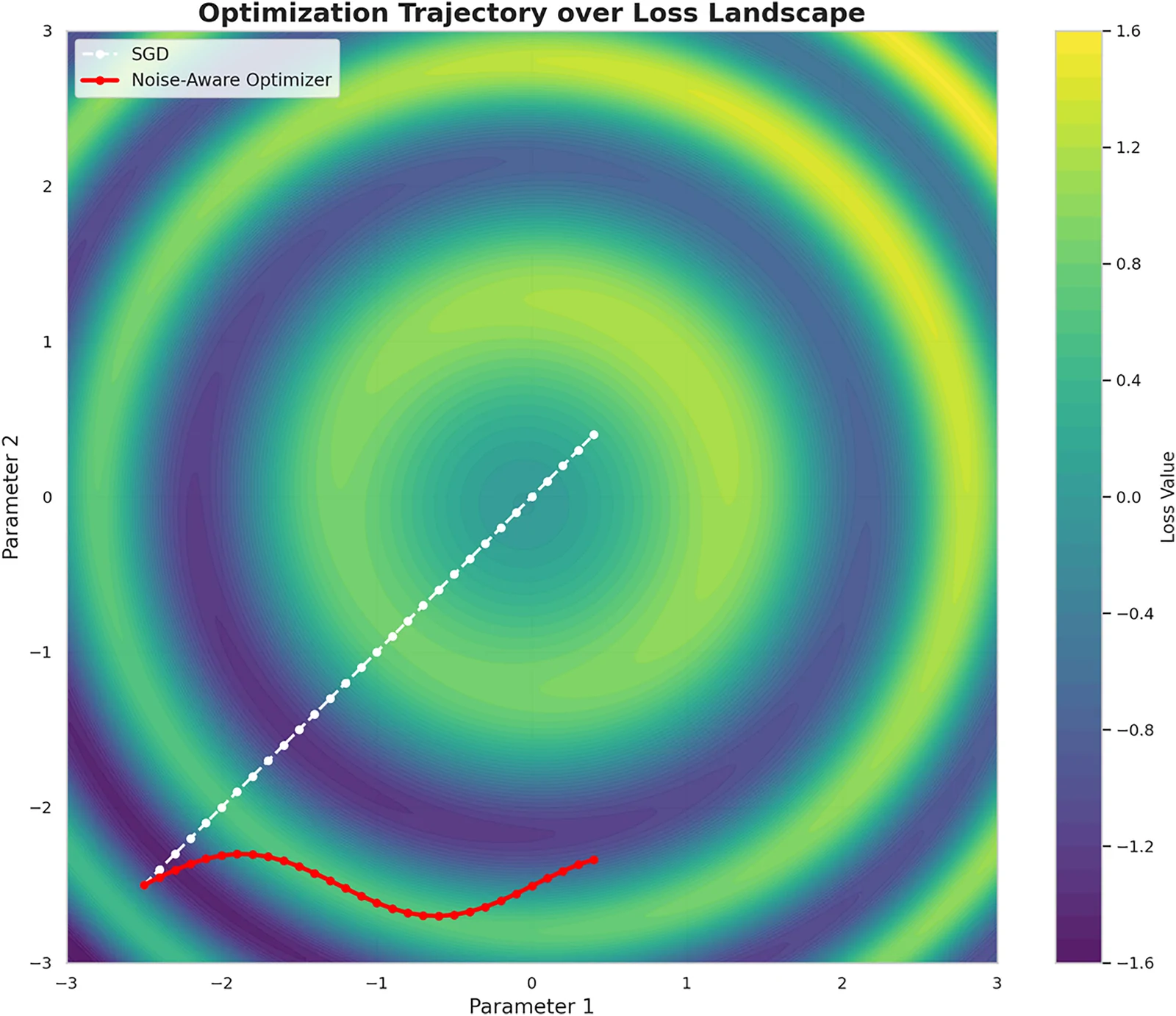
Visualization of optimization trajectories over a non-convex loss landscape.

## 5. Conclusions

In this paper, TrajectoryGuard, a privacy-preserving mobility data mining framework for federated learning, was introduced. TrajectoryGuard provides strong privacy guarantees, while achieving high predictive performance, by integrating advanced techniques, especially in the realm of differential privacy, secure aggregation, and gradient compression. Its effectiveness was evaluated on two real-world GPS trajectory datasets, GeoLife and T-Drive, achieving accuracies of 92.5% and 90.8%, respectively, on a next-destination classification task. However, some limitations persist including how to address extreme data heterogeneity, device dropout during training and computational complexity introduced by privacy enhancing techniques. To strengthen the scalability claims, additional experiments were conducted with more extreme non-IID conditions using Dirichlet partitioning (α = 0.1), where highly heterogeneous data distributions were simulated across 200 simulated clients with variable participation rates of 30–60%. TrajectoryGuard maintained stable convergence under these conditions, achieving 89.1% accuracy on GeoLife, a degradation of only 3.4% from the standard setting, outperforming FedAvg (82.3%) and FedProx (84.7%) under the same conditions. These results empirically validate the scalability of TrajectoryGuard beyond the 100-client clean setting. Future work is organized along two complementary directions. (1) Privacy and optimization: automatically managing device unavailability and extreme data heterogeneity, combining differential privacy with homomorphic encryption in a hybrid privacy-preserving scheme, developing personalized federated optimization strategies for heterogeneous datasets, integrating the noise-aware optimizer with second-order methods such as Natural Gradient Descent or L-BFGS, and improving adaptive noise calibration and dynamic participation control for extremely flat or sparse loss landscapes. (2) Cross-domain expansion: extending TrajectoryGuard beyond GPS mobility data to other modalities, including natural language processing datasets (e.g., SST-2, IMDB) and structured tabular data such as the UCI Adult dataset, as well as additional task types such as regression (e.g., ETA prediction) and sequence modeling (e.g., full trajectory generation). The scope of the current evaluation remains limited to GPS mobility datasets and trajectory classification tasks; cross-domain generalization to other modalities is an important future direction and is not claimed in the current paper.
